# Diet and Anxiety: A Scoping Review

**DOI:** 10.3390/nu13124418

**Published:** 2021-12-10

**Authors:** Monique Aucoin, Laura LaChance, Umadevi Naidoo, Daniella Remy, Tanisha Shekdar, Negin Sayar, Valentina Cardozo, Tara Rawana, Irina Chan, Kieran Cooley

**Affiliations:** 1 Canadian College of Naturopathic Medicine, Toronto, ON M2K 1E2, Canada; dremy@ndnet.ccnm.edu (D.R.); tshekdar@ndnet.ccnm.edu (T.S.); sayar.negin@gmail.com (N.S.); valentinacardozov@gmail.com (V.C.); trawana@ndnet.ccnm.edu (T.R.); irinacchan@gmail.com (I.C.); kcooley@ccnm.edu (K.C.); 2 Department of Psychiatry, McGill University, Montreal, QC H3A 0G4, Canada; laura.lachance@mail.mcgill.ca; 3 St. Mary’s Hospital Centre, Montreal, QC H3T 1M5, Canada; 4 Massachusetts General Hospital, Boston, MA 02114, USA; unaidoo@mgh.harvard.edu; 5 Department of Psychiatry, Harvard Medical School, Boston, MA 02115, USA; 6 Anthrophi Technologies, Toronto, ON M6H1W2, Canada; 7 School of Public Health, Australian Research Centre in Complementary and Integrative Medicine (ARCCIM), University of Technology Sydney, Ultimo 2007, Australia; 8 Pacific College of Health Sciences, San Diego, CA 92108, USA; 9 National Centre for Naturopathic Medicine, Southern Cross University, Lismore 2480, Australia

**Keywords:** nutrition, diet, food, anxiety, mental health, psychiatry, nutritional science, dietetics

## Abstract

Anxiety disorders are the most common group of mental disorders. There is mounting evidence demonstrating the importance of nutrition in the development and progression of mental disorders such as depression; however, less is known about the role of nutrition in anxiety disorders. This scoping review sought to systematically map the existing literature on anxiety disorders and nutrition in order to identify associations between dietary factors and anxiety symptoms or disorder prevalence as well as identify gaps and opportunities for further research. The review followed established methodological approaches for scoping reviews. Due to the large volume of results, an online program (Abstrackr) with artificial intelligence features was used. Studies reporting an association between a dietary constituent and anxiety symptoms or disorders were counted and presented in figures. A total of 55,914 unique results were identified. After a full-text review, 1541 articles met criteria for inclusion. Analysis revealed an association between less anxiety and more fruits and vegetables, omega-3 fatty acids, “healthy” dietary patterns, caloric restriction, breakfast consumption, ketogenic diet, broad-spectrum micronutrient supplementation, zinc, magnesium and selenium, probiotics, and a range of phytochemicals. Analysis revealed an association between higher levels of anxiety and high-fat diet, inadequate tryptophan and dietary protein, high intake of sugar and refined carbohydrates, and “unhealthy” dietary patterns. Results are limited by a large percentage of animal and observational studies. Only 10% of intervention studies involved participants with anxiety disorders, limiting the applicability of the findings. High quality intervention studies involving participants with anxiety disorders are warranted.

## 1. Introduction

The term anxiety describes the experience of worry, apprehension, or nervousness in association with physical, cognitive, and behavioral symptoms. Anxiety may be experienced occasionally as part of normal life and may be adaptive if it increases preparedness for novel situations. If anxiety symptoms are persistent, excessive, or interfere with functioning, they can become pathological [1].

Several anxiety disorders have been defined. Generalized anxiety disorder involves excessive worry in multiple domains and associated physical symptoms that are present for at least six months leading to clinically significant distress or impairment in functioning [1]. Panic disorder is characterized by unexpected and recurrent panic attacks and at least one month of persistent worry about having a subsequent panic attack or significant behavior changes related to the attack [2]. Agoraphobia involves feelings of intense fear of situations or spaces where escape may be difficult or help may not be available in the event or panic or other incapacitating symptoms [3]. Social anxiety disorder involves marked anxiety and fear of a social situation where an individual is exposed to possible scrutiny by others [4]. Specific phobia is an excessive fear of specific object or situation [5].

Anxiety disorders exert a significant burden at both an individual and societal level. Individuals with anxiety disorders report a high degree of psychological distress, significant disability [6] and a reduction in quality of life [7]. The presence of an anxiety disorder is associated with higher use of both primary care, emergency room visits, and specialist healthcare services [8]. These disorders are also highly prevalent. The national comorbidities study established the lifetime prevalence of any anxiety disorder at 31.2%, the highest of any category of psychiatric illnesses [9].

The treatment approaches most frequently used in the management of anxiety disorders are psychotherapy and psychopharmacology [10]. While many patients find these therapies beneficial, a significant number of individuals report that these treatment options are not accessible, tolerable, or effective in providing adequate relief of anxiety symptoms [11]. For these reasons, there is interest in the evaluation of adjunctive or alternative therapeutic approaches.

Nutritional psychiatry is an emerging field of study related to the use of nutritional interventions in the prevention and treatment of mental health disorders. Despite increasing evidence of beneficial effects, nutritional recommendations are provided to psychiatric patients infrequently in clinical practice. Recently, high quality intervention studies have demonstrated an antidepressant effect of nutritional interventions [12,13]. However, the amount of research on anxiety disorders lags behind that of mood disorders [14,15]. There is a clear lack of studies delivering diet counselling, education, or food as an intervention to individuals with diagnosed anxiety disorders as well as systematic synthesis of the existing literature on the relationship between dietary factors and anxiety symptoms or disorders. The objective of the present review was to systematically map out the body of existing literature on anxiety symptoms/disorders and nutrition in order to identify nutritional factors associated with higher or lower levels of anxiety and to identify knowledge gaps and opportunities for further research.

## 2. Materials and Methods

The review followed established methodological approaches for scoping reviews using the framework presented by Arskey and O’Malley for the conduct of scoping reviews [16]. Scoping reviews aim to identify and describe the breadth of literature on a topic when it is either highly complex, involves a broad array of study designs, or when a comprehensive review is being completed for the first time; all of these factors apply to the present review. Scoping reviews aim to map key concepts in a field of study and the available types of evidence. The review is completed in a way that is systematic, highly rigorous, and transparent in order to minimize bias. The protocol used in the present study was adapted from a similar project completed by the authors on the topic of diet and psychosis [17].

An extensive *a priori* search strategy was developed and executed with the guidance of an experienced medical librarian. Using the Ovid platform, we searched Ovid MEDLINE^®^, including Epub Ahead of Print, In-Process & Other Non-Indexed Citations, and Embase Classic + Embase. We used controlled vocabulary (e.g., “Anxiety Disorders”, “Nutritional Physiological Phenomena”, “Food”) and keywords (e.g., anxiety, nutrition, diet). We adjusted vocabulary and syntax as necessary across the databases. There were no language or date restrictions on any of the searches, but we removed opinion pieces (e.g., editorials) from the results. We performed the searches on 25 March 2020. The full search, as executed, is available in Supplemental File S1.

Screening of abstracts and titles was completed using the online open-source program Abstrackr [18] which allowed for concurrent and blind duplicate screening as well as tagging by dietary constituent. Manual screening was completed until the program’s artificial intelligence predicted the presence of additional relevant studies as unlikely. Previous testing of this program has demonstrated that the likelihood of missing relevant studies is very low [18,19,20]. As an extra precaution, once screening had reached the point where Abstrackr’s prediction score reported ‘zero additional studies likely to be relevant’, and no studies were being identified as being relevant, an additional 100 articles were screened in each section before screening was stopped. Screening of abstracts and titles was completed in duplicate. Disagreement was resolved by consensus.

Studies were eligible for inclusion if they involved the evaluation of changes in the level of anxiety symptoms or the presence/absence of anxiety disorders in humans or animal models as well as assessing or modifying a component of participant diet. This included assessment or modification of dietary patterns, individual foods, supplements, or natural health products that provide an active constituent naturally occurring in the general North American diet. Studies were ineligible if they assessed or administered herbal medicines (apart from those used for culinary purposes in the general North American diet) or constituents which are not typically found in significant quantities in the human diet (i.e., St. John’s Wort, GABA, or S-adenosylmethionine) or if they assessed levels of endogenously produced dietary components (i.e., cholesterol, vitamin D, or non-essential amino acids) in the absence of supplementation or measurement of intake. Eligible study designs included human observational and experimental studies, animal studies, and meta-analyses. Studies were excluded if they assessed the impact of maternal diet on offspring anxiety levels. Review articles, opinion papers, letters, and systematic reviews (without meta-analysis) were excluded, as were non-English language papers or inaccessible papers in cases where the abstract contained insufficient information for data extraction.

Full text screening was completed concurrently with data extraction. Data extraction was completed using piloted extraction templates developed for a similar scoping review conducted by the study authors and double checked by MA for accuracy [17]. Analysis was completed by sorting the studies with common interventions and methodology types and counting the number of studies reporting an association with increased or decreased anxiety symptoms/disorders, or no association. These data were used to create figures that communicate an overview of the evidence on each topic. Studies reporting a statistically significant improvement in at least one subpopulation or measure of anxiety symptomatology were categorized as “associated with decreased anxiety”. Studies reporting an increase in anxiety symptoms or prevalence in at least one subpopulation or measure of anxiety symptomatology were categorized as “associated with increased anxiety”. Studies that reported no significant change in anxiety symptoms or prevalence were categorized as “no association with anxiety”. A small number of studies which reported mixed findings such as a combination of increased and decreased anxiety symptoms were not included in the figures. In order to allow concise display and the comparison of all studies, the number of studies reporting an association between improved symptoms with higher intake of a nutrient were combined with studies reporting and association between worse symptoms with lower intake of the nutrient. Counts are depicted in figures. The figures are oriented so that they report the relationship between higher intake of the diet constituent with anxiety. Within each section, a narrative summary was completed to highlight trends, gaps, and areas that warrant further study. When available, narrative summaries also reported on proposed mechanisms and safety. Finally, we created a list of dietary factors that, based on the review findings, may to be associated with less anxiety and more anxiety symptoms/disorders. This process of categorization was done based on the following criteria related to the volume and consistency of evidence. Dietary factors were included in these two categories when there were at least five studies reporting on the relationship with anxiety, and the majority of the data points (>60%) showed a consistent association. These criteria were developed post hoc as the volume, and consistency of the evidence was unknown at the time of protocol development.

## 3. Results

### 3.1. Search Results

The search identified 55,914 unique results that were screened in two phases: by title/abstract and by full text. The study authors manually screened 13,286 results while Abstrackr’s artificial intelligence screened the remaining results. Following title and abstract screening, 2213 articles were included.

**Figure 1:**
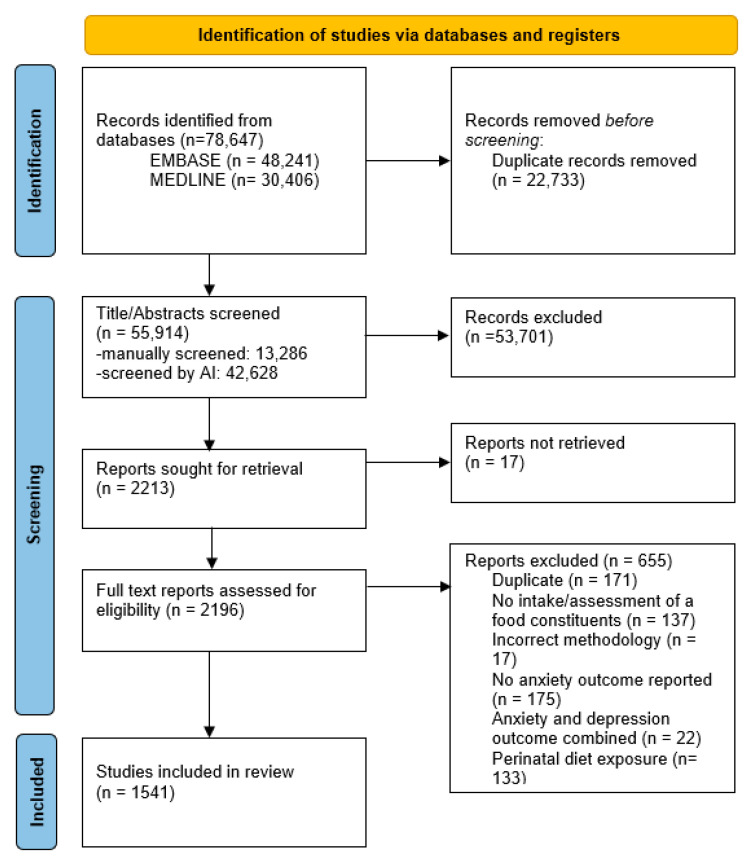
PRISMA flow diagram. AI: artificial intelligence.

Seventeen articles could not be retrieved in full text. During full text screening, an additional 655 studies were excluded (Supplemental File S2). 1541 studies were included in the final data analysis (Figure 1, Supplemental File S3).

### 3.2. Study Characteristics

**Figure 2:**
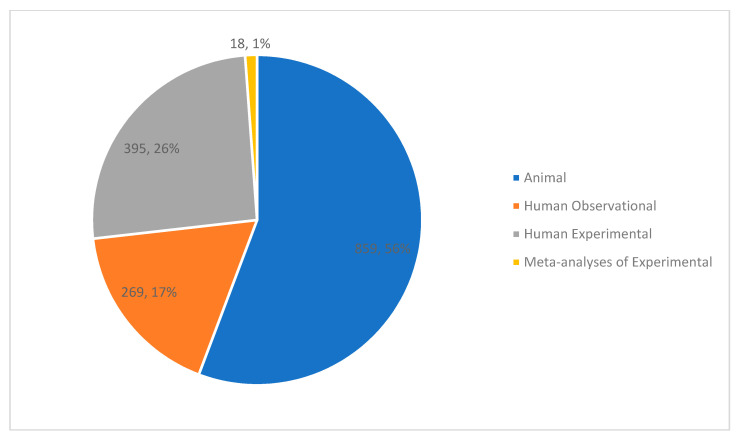
Distribution of included studies by methodology (count, percent).

More than half of the studies in our analysis were conducted using animal models (*n* = 859) (Figure 2). The animal studies were primarily conducted in rodents (97%) with the remaining 3% of studies conducted in zebrafish, pigs, lemurs, monkeys, cats, horses, and tilapia.

**Figure 3:**
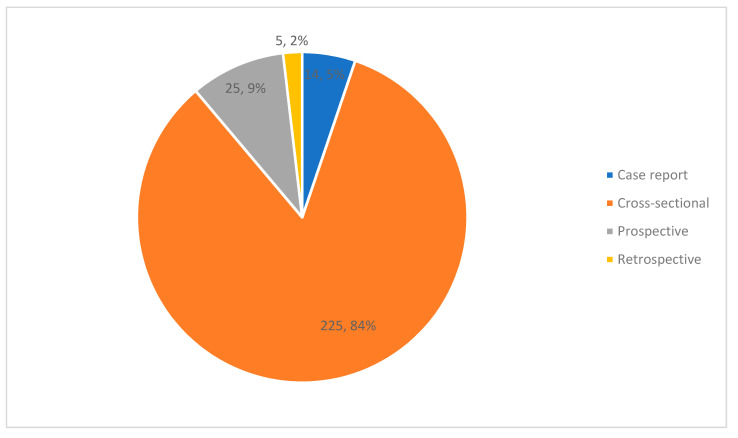
Types of observational studies (count, percent).

The observational studies included 14 case reports; in 11 reports, subjects had anxiety disorders or elevated anxiety symptoms at baseline (Figure 3). In total, the reports described 44 individual cases. An additional 255 publications described cross-sectional, prospective, or retrospective observational studies. One meta-analysis of observational studies was conducted [21]. Of the observational studies, 88% were cross-sectional in design and 13 studies (5%) specifically included individuals with anxiety disorders or elevated anxiety symptoms. Nutrient intake was assessed in 201 studies while nutrient levels, in various body tissues, were measured in 40 studies. Sample size varied widely, from 14 to 296,121 participants (Mean: 6315.7, SD: 28,423.9).

**Figure 4:**
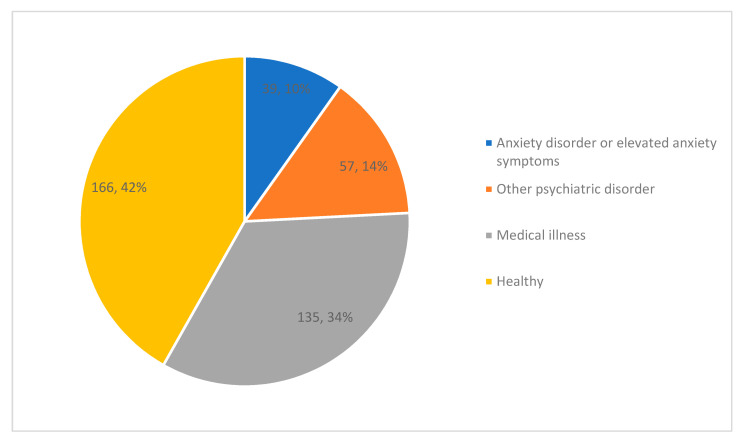
Participant populations in human experimental studies (count, percent).

Regarding experimental studies, 395 met criteria for inclusion as well as an additional 18 meta-analyses of experimental studies. Of the individual studies, the number of participants ranged from 3 to 2730 (Mean: 99.3, SD: 198.3). Of the 395 trials, 335 (85%) included a comparison arm, 312 (79%) utilized randomization, and 23 (61%) utilized blinding. Thirty-nine trials (10%) included participants with anxiety disorders or elevated anxiety symptoms. An additional 57 trials assessed anxiety in participants with other psychiatric illnesses while the remaining studies included participants with medical illnesses or healthy participants (Figure 4). Excluding the studies that assessed the immediate impact of food on anxiety symptoms (*n* = 72), the average duration of the experimental studies was 15.8 weeks (SD: 18.3 weeks). Most of the studies (*n* = 331) provided the dietary intervention without co-interventions. An exercise co-intervention was delivered in 32 studies, while 20 included a psychosocial component and ten co-administered a medication. Sixty-nine percent of experimental studies identified a primary outcome related to mental health.

### 3.3. Dietary Patterns

**Figure 5:**
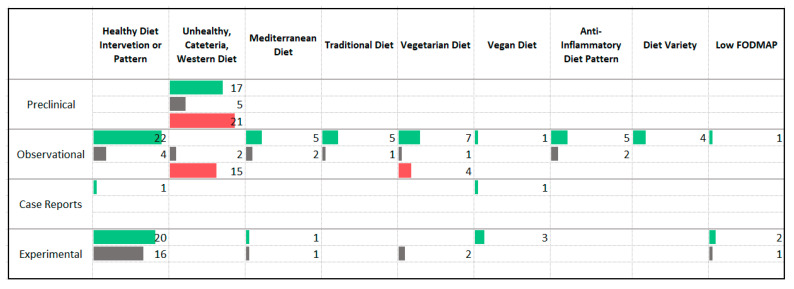
Studies assessing the composition of dietary patterns. ⯀ Higher intake or levels associated with decreased anxiety. ⯀ No association between intake or levels and anxiety. ⯀ Higher intake or levels associated with increased anxiety.

**Figure 6:**
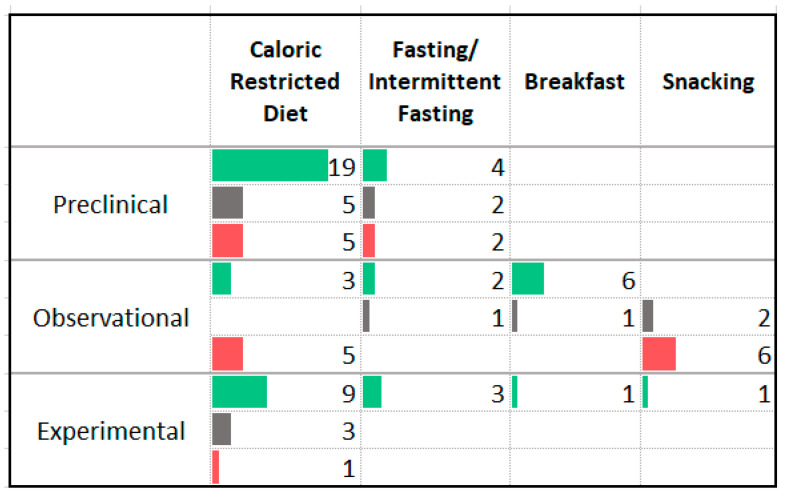
Studies assessing the amount of food consumed or timing of eating. ⯀ Higher intake or levels associated with decreased anxiety. ⯀ No association between intake or levels and anxiety. ⯀ Higher intake or levels associated with increased anxiety.

Many studies were identified that assessed the impact of dietary patterns on anxiety symptoms severity or anxiety disorder prevalence including both animal models (*n* = 101) and human studies with observational (*n* = 102) and experimental (*n* = 84) designs. Dietary pattern studies evaluated the impact of combinations of foods or patterns of eating. Studies looked at both the types of foods that were consumed (Figure 5) and the quantity and timing of food consumption (Figure 6). Because studies may have defined specific dietary patterns differently, it is noted that significant heterogeneity exists within each category. In general, “Healthy” diet patterns were described as diets aligned with generally accepted principles of healthy eating. Many involved the calculation of a healthy eating score or index and were defined by higher intake of vegetables, fruit, whole grains, fish, legumes, and unprocessed meat. Diets or dietary patterns defined as “Unhealthy” or “Western” generally included higher intake of processed foods, sugar and sweetened foods, soft drinks, fried foods, processed meats, “junk food”, and “fast food”. In the animal studies, the “Western” or “Cafeteria” diet included a combination of high fat and high carbohydrate, in particular, high saturated fat and high refined carbohydrates. In many cases, the diet was designed to be highly palatable and to induce obesity [22,23].

The animal studies reported a mixture of anxiogenic and anxiolytic effects following administration of the “unhealthy”, “cafeteria”, or “Western” style eating patterns. Predominantly anxiolytic effects were seen following caloric restriction and fasting.

Observational studies showed an association between lower anxiety symptom severity or disorder prevalence and “healthy” diet patterns, the Mediterranean diet, traditional diets, the vegetarian diet, consumption of breakfast, anti-inflammatory diet patterns, and increased diet variety. Higher anxiety symptom severity or disorder prevalence was associated with “unhealthy” diet patterns, caloric restriction, and snacking. It is noted that among the observational studies assessing the relationship between vegetarianism and anxiety symptoms, two of the three studies that reported an increase in anxiety were prospective in design while all of the studies reporting an association between the diet pattern and less anxiety were cross-sectional in design. Two case reports described improvement in anxiety symptom severity following multimodal interventions with a dietary component. One involved the elimination of “inflammatory foods” in combination with exercise and psychological treatment [24]. The other delivered a vegan diet in combination with fruit and vegetable juice, nutritional supplements, exercise and stress management techniques [25].

Of the experimental studies delivering an intervention that promoted healthy eating behaviors, twenty reported a reduction in anxiety symptoms while sixteen did not observe a significant effect. Only two of these trials recruited individuals with anxiety disorders [26,27]; the remaining studies recruited individuals with medical illnesses or healthy participants. One randomized controlled trial enrolled participants with moderate to severe anxiety disorders and assessed the impact of dietary counselling, in combination with a multivitamin and a herbal remedy, compared to a psychosocial intervention, and reported a significant improvement in anxiety symptoms with the combination intervention as compared to the psychosocial intervention [26]. Another study randomized individuals with anxiety and/or depressive disorders to either dietician consultations or an attention control intervention; significant improvement was seen in both treatment groups [27]. Nine of thirteen studies promoting caloric restriction reported an improvement in anxiety symptoms. While one of the studies recruited participants with elevated anxiety symptoms [28], all involved overweight and obese participants. A meta-analysis of studies delivering caloric restriction to adults with obesity did not find a significant reduction in anxiety symptoms [29] while a meta-analysis on the same topic that included child and adolescent populations did report a reduction in anxiety [30].

### 3.4. Carbohydrates

**Figure 7:**
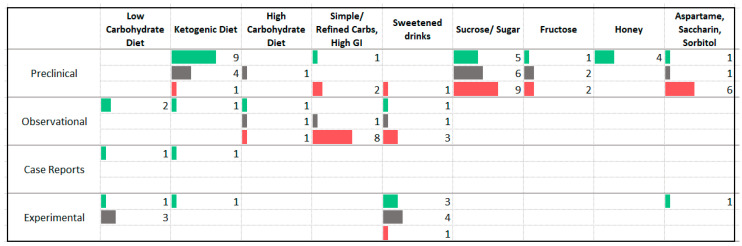
Studies assessing dietary carbohydrates and anxiety. ⯀ Higher intake or levels associated with decreased anxiety. ⯀ No association between intake or levels and anxiety. ⯀ Higher intake or levels associated with increased anxiety.

One trend that was observed among the carbohydrate studies was a relationship between higher intake of simple or refined carbohydrates, higher glycemic index diet, or sugar intake, and higher levels of anxiety (Figure 7). This association was reported by several animal and observational studies. Similarly, 75% of the 12 animal studies and the only human experimental trial assessing the impact of artificial sweeteners (aspartame, saccharin, and sorbitol) reported an increase in anxiety symptoms. One observational study reported on the relationship between fiber and anxiety; at long term follow up two to three years after completing a program aimed at increasing fiber intake, 14 irritable bowel syndrome patients reported lower anxiety symptoms [31].

Very limited research has been undertaken in the form of human experimental studies that sought to reduce carbohydrate intake (*n* = 4); none included participants with anxiety disorders. Of four studies, one study involving obese or overweight participants reported an improvement in anxiety symptoms following a low carbohydrate diet. Eight studies evaluated the effect of consuming sweetened drinks on anxiety in humans. These studies administered carbohydrate-rich drinks to patients undergoing surgical procedures and measured the immediate effects on anxiety symptom severity, often in comparison with pre-operative fasting. The findings of these studies were mixed.

### 3.5. Protein

**Figure 8:**
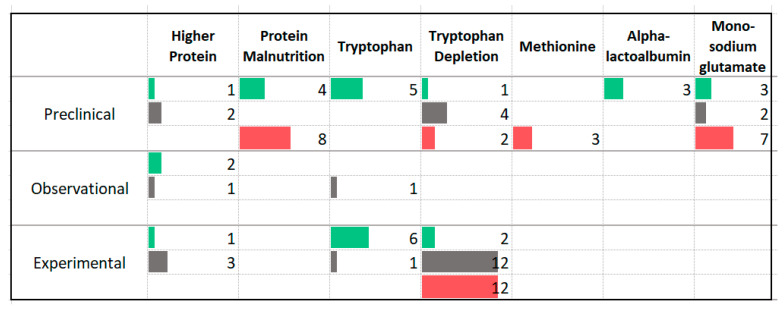
Studies assessing protein and anxiety. ⯀ Higher intake or levels associated with decreased anxiety. ⯀ No association between intake or levels and anxiety. ⯀ Higher intake or levels associated with increased anxiety.

Limited research has investigated the effects of different levels of dietary protein on anxiety symptom severity or disorder prevalence (Figure 8). Two thirds of the 12 animal studies assessing the impact of protein malnutrition reported a worsening of anxiety symptoms. Four human experimental studies delivered a high protein diet; three studies, involving participants without anxiety disorders, reported no effect on anxiety symptom severity. One experimental study involving three participants with elevated anxiety symptoms reported improvement following a high protein, low carbohydrate diet [32]. A very small amount of research has been completed comparing plant and animal sources of protein. One observational study reported a benefit with higher intake of animal protein [33], the other reported no difference [34]. The food additive monosodium glutamate (MSG), a modified amino acid, was associated with increased levels of anxiety in animal models.

There was an association between higher anxiety symptoms and tryptophan depletion, among human experimental studies. Supplementation of tryptophan resulted in decreased anxiety symptoms in both animal and experimental studies. Of the studies assessing the effect of tryptophan depletion, 10 involved individuals with anxiety disorders; five reported no effect while five reported a worsening of anxiety symptoms. Of the studies administering a tryptophan supplement, two involved individuals with anxiety disorders [35,36]; both studies reported improvement.

### 3.6. Fats

**Figure 9:**
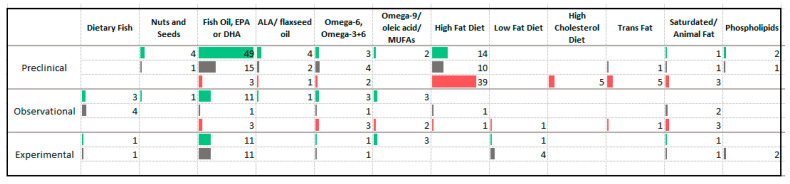
Studies assessing dietary fats and anxiety. ⯀ Higher intake or levels associated with decreased anxiety. ⯀ No association between intake or levels and anxiety. ⯀ Higher intake or levels associated with increased anxiety.

Compared with other categories of nutrients, a large number of animal studies have investigated the effects of increased intake of fat on rodent models of anxiety (Figure 9). Many animal studies (*n* = 39) have reported an anxiogenic effect of a high fat diet. A smaller number have reported a similar effect from diets high in cholesterol (*n* = 5) and trans fats (*n* = 6). A large number of animal studies have reported anxiolytic effects of omega-3 fatty acids (*n* = 35), Docosahexaenoic acid (*n* = 9), Eicosapentaenoic acid (*n* = 3), and alpha-linolenic acid (*n* = 4). Human observational studies reported an association between higher intake of omega-3 fatty acids and lower levels of anxiety.

Twenty-two human experimental studies have measured changes in anxiety symptoms following supplementation with omega-3 fatty acids. Eleven studies reported an improvement in anxiety symptoms, the remaining studies were equivocal. Two of three meta-analyses of trials delivering omega-3 fatty acid supplements reported benefit to anxiety symptoms [37,38,39]. While nine of the experimental studies included participants with psychiatric disorders such as eating disorders, substance use disorders and ADHD, only one trial involved participants with anxiety disorders [40]. In this trial, researchers provided polyunsaturated fatty acids (omega-3 and omega-6) to 126 sufferers of test anxiety and reported an improvement in symptom severity after three weeks.

### 3.7. Vitamins

**Figure 10:**
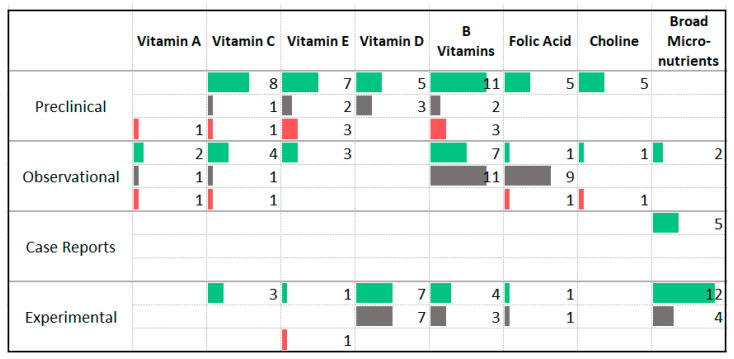
Studies assessing vitamins and anxiety. ⯀ Higher intake or levels associated with decreased anxiety. ⯀ No association between intake or levels and anxiety. ⯀ Higher intake or levels associated with increased anxiety.

A large number of animal studies (*n* = 56) have investigated the effects of vitamins B, C, D, E, choline, and folic acid and reported primarily anxiolytic effects (Figure 10). Among the human observational studies, the majority of studies assessing vitamin C, vitamin E, and broad-spectrum micronutrients reported an association with less anxiety symptoms while the majority of the studies assessing levels or intake of B vitamins and folic acid reported no association with anxiety symptom severity. Additionally, five case reports reported improvement in anxiety symptoms following broad-spectrum micronutrient supplementation.

Among the human experimental studies, several studies reported improved anxiety symptoms following supplementation of vitamin C (*n* = 3) and broad-spectrum micronutrients (*n* = 12); mixed findings were reported by studies assessing vitamin D, B vitamins, and folic acid. Two human experimental studies involved participants with anxiety disorders. These trials delivered vitamin D [41] and two different broad-spectrum vitamin and micronutrient formulas [42], one assessed at a low and high dose. Both studies reported a reduction in anxiety symptoms.

A number of studies were identified that delivered combination natural health products to human (*n* = 22) or animal (*n* = 11) participants. The formulas included combinations of vitamins (typically two or three) or combinations of fatty acids, vitamins, minerals, probiotics, and amino acids. Among the human studies, 16 of 22 reported a significant improvement in anxiety symptoms. Among the animal studies, eight of 11 reported a significant improvement. The remaining studies reported no change. Two human trials involved participant with anxiety disorders or elevated anxiety symptoms. One included individuals with GAD [43], one included individuals with high trait anxiety levels [44]. The former delivered a combination of three vitamins (A, C, and E) and reported an improvement. The latter delivered a combination of probiotics, B vitamins and proteins and did not find a reduction in anxiety symptoms; however, other self-reported and biological markers of stress improved.

### 3.8. Minerals

**Figure 11:**
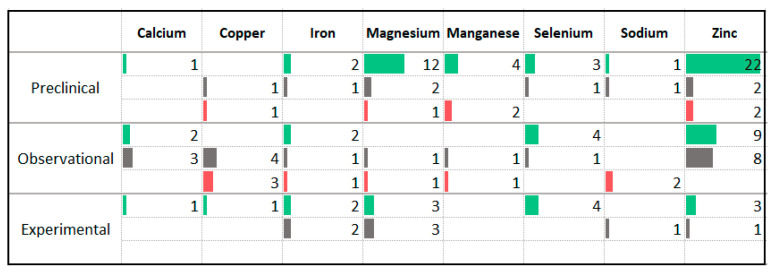
Studies assessing minerals and anxiety. ⯀ Higher intake or levels associated with decreased anxiety. ⯀ No association between intake or levels and anxiety. ⯀ Higher intake or levels associated with increased anxiety.

The minerals that were assessed for an impact on anxiety symptoms in animal models most frequently are zinc, magnesium, manganese, and selenium (Figure 11). The animal studies reported a largely consistent anti-anxiety effect from supplementing these nutrients. The observational studies assessing tissue levels of magnesium most frequently reported no association with anxiety levels or anxiety disorder prevalence. Observational studies assessing tissue levels of zinc reported a combination of protective effects and no association while the observational studies assessing selenium primarily reported an association between higher intake and lower symptom severity. A small number of observational studies reported an association between higher copper or sodium and increased anxiety symptoms or disorder prevalence.

A limited number of intervention studies have delivered minerals in supplemental form, including selenium, zinc, magnesium, iron, copper, and calcium. All four studies that administered selenium supplements reported improvement in anxiety symptoms. Studies administering other minerals reported a mixture of anti-anxiety effects and no effect. Only one experimental study included participants with anxiety disorders [45]. This open label study provided zinc to 38 participants for eight weeks and reported an improvement in anxiety symptoms.

### 3.9. Vegetables and Fruit

**Figure 12:**
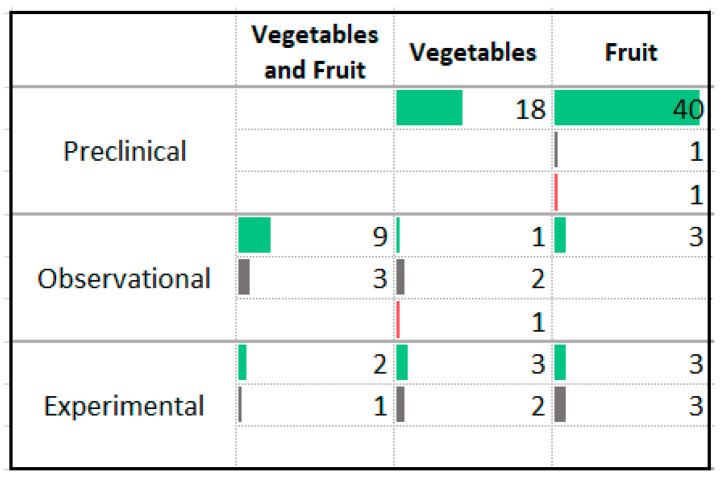
Studies assessing vegetables and/or fruit and anxiety. ⯀ Higher intake or levels associated with decreased anxiety. ⯀ No association between intake or levels and anxiety. ⯀ Higher intake or levels associated with increased anxiety.

The results of the studies related to vegetables and fruit were largely positive (Figure 12). The majority (64%, *n* = 60) of the studies were conducted in animal models. These trials involved the administration of single fruits or vegetables and 58 of 60 reported a reduction in anxiety symptom severity. The most commonly studied foods included citrus fruits (*n* = 13), grape (*n* = 7), berries (*n* = 6), pomegranate (*n* = 6), fennel (*n* = 4), and lettuce (*n* = 4) with a wide variety of vegetables and fruits used in the remaining studies. Several observational studies reported an association between higher fruit and vegetable intake and lower levels of anxiety symptoms or disorder prevalence. A small number of intervention studies increased intake of individual vegetable or fruits; studies delivering cherries, tomato juice, orange juice, and fennel reported reduction of anxiety symptom severity. Three experimental studies delivered interventions aimed at increasing intake of vegetable and/or fruit servings consumed by participants [46,47,48]. Two reported a decrease in anxiety symptoms. None of the human experimental studies involved participants with anxiety disorders.

### 3.10. Phytochemicals

**Figure 13:**
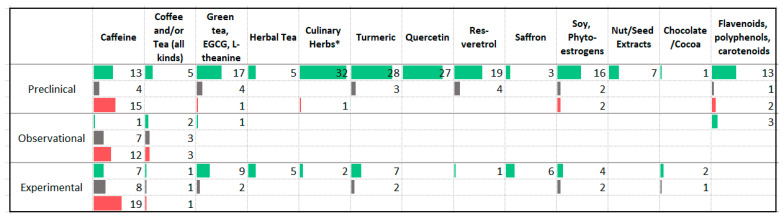
Studies assessing phytochemicals and anxiety * Culinary Herbs: rosemary, cinnamon, coriander, basil, nigella; Herbal tea: chamomile, hibiscus, rose tea. ⯀ Higher intake or levels associated with decreased anxiety. ⯀ No association between intake or levels and anxiety. ⯀ Higher intake or levels associated with increased anxiety.

A large number of studies have explored the effects of caffeine on anxiety symptoms (Figure 13). Many of these animal (*n* = 37) and human experimental (*n* = 33) studies administered caffeine as a supplement or provided energy drinks to subjects. While the animal studies reported a mixture of anxiogenic and anxiolytic effects, the observational and human experimental studies were more likely to report worsening anxiety symptoms with higher intake of caffeine. Several animal and human experimental studies assessed the impact of green tea and its constituents epigallocatechin-3-gallate (EGCG) and l-theanine on anxiety levels. The studies reported primarily anti-anxiety effects; however, a meta-analysis of trials delivering L-theanine and EGCG failed to detect a significant benefit [49].

Anti-anxiety effects have been reported in animal studies assessing a wide range of plants and plant constituents that may be found in the North American diet with primarily anxiolytic findings reported. These included culinary herbs (rosemary, cinnamon, coriander, basil, and nigella), herbal teas (chamomile, hibiscus, and rose tea), the phytonutrients curcumin (found in turmeric), quercetin (found in various vegetables and fruits), resveratrol (found in grapes), saffron and its constituents, soy and its constituents and other phytoestrogenic foods, nut and seed extracts, chocolate and cocoa and a variety of flavonoids, polyphenols, and carotenoids. Additionally, human experimental studies delivering green tea, curcumin, saffron, chamomile, and soy also reported anti-anxiety effects. Meta-analyses of experimental studies using chamomile [50], saffron [51], and curcumin [52,53] found a decrease in anxiety symptoms, while the meta-analysis of studies administering resveratrol reporting a non-significant improvement [54]. A small number of the human experimental studies involved participants with anxiety disorders including three studies using chamomile [55,56,57], two using saffron [58,59], one using curcumin [60], and one using l-theanine [61]. All trials, with the exception of the l-theanine study [61], reported a reduction in anxiety symptoms severity.

### 3.11. Food Allergy and Intolerance

**Figure 14:**
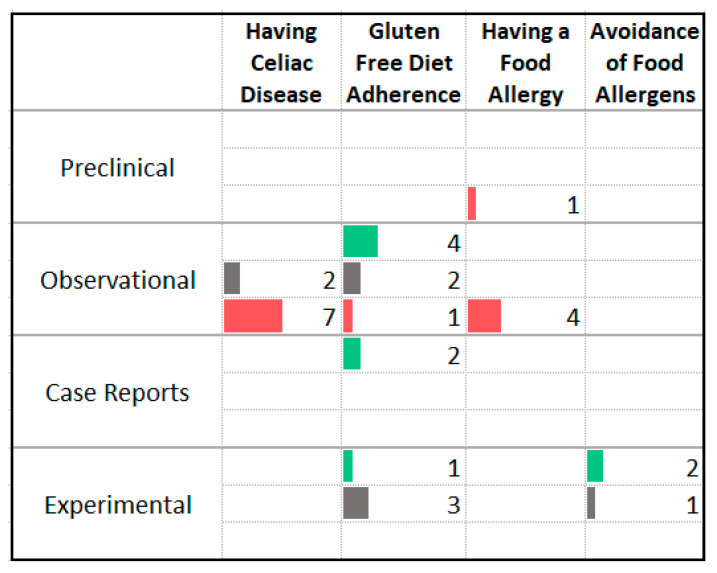
Studies assessing food allergies or sensitivities and anxiety. ⯀ Higher intake or levels associated with decreased anxiety. ⯀ No association between intake or levels and anxiety. ⯀ Higher intake or levels associated with increased anxiety.

A small body of evidence, primarily observational in nature, suggests a possible connection between food allergy or intolerance and anxiety symptoms (Figure 14). Eleven of thirteen studies found higher levels of anxiety symptoms in participants with celiac disease or a food allergy. Consumption of a gluten-free diet or avoidance of other food allergens was associated with improved anxiety symptoms in observational studies and two case reports. Seven experimental studies have assessed these diet interventions with three reporting benefit. Participants were predominantly irritable bowel syndrome sufferers; none included individuals with anxiety disorders.

### 3.12. Gut Microbiome

**Figure 15:**
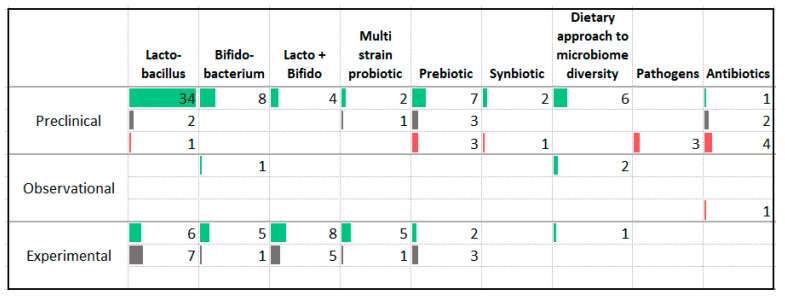
Studies assessing the microbiome and anxiety. ⯀ Higher intake or levels associated with decreased anxiety. ⯀ No association between intake or levels and anxiety. ⯀ Higher intake or levels associated with increased anxiety.

A number of studies have assessed the impact of probiotic and prebiotic supplementation in animals and humans (Figure 15). Animal trials have reported significant improvement in anxiety symptoms with supplementation of *Lactobacillus* strains, *Bifidobacterium* strains, combinations of *Lactobacillus* and *Bifidobacterium* strains, prebiotics, and synbiotics (combinations of prebiotics and probiotics). Interventions aimed at increasing microbiome diversity or addition of fermented foods or fiber were associated with decreased anxiety symptom severity. Administration of pathogenic organisms and antibiotics were associated with worsening anxiety symptom severity.

Among the human studies, predominantly positive findings were reported by trials administering *Bifidobacterium* strains and multi-strain probiotics while trials administering *Lactobacillus* strains, combinations of *Lactobacillus* and *Bifidobacterium* strains, and prebiotics reported either positive findings or no effect. Two trials involved participants with anxiety disorders [62,63]; one reported a benefit [63]. Three meta-analyses have pooled the trials assessing anxiety outcomes following probiotic supplementation [64,65,66]; one of three reported a significant improvement [65].

## 4. Discussion

The results of this scoping review suggest a possible association between more or less anxiety and a range of dietary constituents and patterns. Table 1 presents a summary of the associations identified in this review.

### 4.1. Dietary Patterns

Overall, there is evidence that certain dietary patterns may influence the development and progression of anxiety disorders. The diets associated with lower anxiety include “healthy” diet patterns, the Mediterranean diet, traditional diets, the anti-inflammatory diet, and diets with increased variety. All of these diet patterns share common elements such as an emphasis on vegetables, fruit, limited sugar and refined grains, and greater consumption of minimally processed foods. However, interpretation of dietary patterns studies is somewhat hindered by the dissimilar definitions used for dietary patterns. For example, in the studies delivering “Western” style high fat/high sugar diets meant to induce obesity, a variety of dietary fats were used. The fats used to supplement some of the high fat diets included lard [67], fish oil [68], soybean oil [69] while other studies specified the percentage fat in the diet but not the type of fat that was added to achieve this amount [70,71,72]. Because the impact of different fatty acids on health outcomes can be highly different [73], categorizing diets as low or high in fat may result in heterogenous findings as a result of the type of fat delivered. Similarly, definitions of “healthy” diets have changed over time [74], potentially contributing to the heterogeneity of study results. Unfortunately, many studies lacked clear definitions of “healthy” or “unhealthy” diet patterns and interventions.

The outcomes associated with the vegetarian or vegan diet were generally positive although somewhat mixed and limited by being largely observational in design. The mixed findings may be due to a variety of factors. There is documentation of the adoption of a vegetarian or vegan diet following the development of an eating disorder [75]. Given the association between eating disorders and anxiety disorders [76], this may explain the association between vegetarianism and higher anxiety found in some observational studies. Furthermore, vegan diets, without adequate supplementation, may lack certain essential nutrients shown to play a role in anxiety disorders such as vitamin B12 [77] and long chain omega-3 fatty acids (EPA and DHA) [78]. Bioavailability of certain nutrients such as iron differ between plant and animal sources, possibly limiting nutrient absorption in vegan diets.

There is a significant lack of human intervention studies involving participants with anxiety disorders or elevated baseline anxiety symptoms. Many studies employed dietary patterns that were indicated for the other medical concerns of the study participants. For example, studies delivered the Low FODMAP diet to participants with irritable bowel syndrome and hypocaloric diets to participants with obesity and assessed changes in anxiety as secondary outcomes. The mechanisms by which dietary patterns impact anxiety symptoms may be the result of a combination of the mechanistic factors discussed in the following sections.

### 4.2. Carbohydrates

The findings of the carbohydrate studies suggest that high intake of sugar and refined carbohydrates may contribute to anxiety symptoms; however, a large proportion of trials are cross-sectional in design, preventing conclusions about causation. There is a need for intervention studies that assess the impact of differing levels of carbohydrate intake in participants with anxiety disorders.

With respect to mechanism, there is evidence that healthy blood sugar regulation is an important factor in mental wellbeing [79]. This relationship may explain the associations seen in the present review between factors that improve blood sugar regulation and lower levels of anxiety symptoms. These include lower intake of sugar and refined carbohydrates, higher fiber intake, regular meals, and caloric restriction.

### 4.3. Protein

The evidence related to the role of protein in anxiety symptoms is preliminary. There is some evidence suggesting that adequate dietary protein and, in particular, adequate tryptophan, may be important in improving anxiety symptoms. Amino acids serve as the building blocks for neurotransmitter synthesis, with tryptophan needed for the production of serotonin [80]. The established role of serotonin in the pathogenesis of anxiety disorders [81] may explain the potential harm associated with inadequate dietary protein and tryptophan. This evidence is strengthened by the involvement of many participants with diagnosed anxiety disorders in the intervention studies included in the present review. The human experimental studies used doses of tryptophan ranging from 250 mg per day from a food source (squash seeds) to 3 g per day as a supplement. Although these doses are considered to be below the level associated with side-effects [82], the trial that administered 3 g per day reported side effects such as itching, nausea and urinary changes [83]. Tryptophan supplements should not be used in combination with serotonergic medications such as SSRI/SNRI due to the possible risk of precipitating serotonin syndrome [84]. Food sources of tryptophan include egg, soy, seeds, fish and meat [85].

### 4.4. Fats

Overall, there is significant animal and human evidence that adequate or supplemental omega-3 fatty acids may have anti-anxiety effects. There is early evidence, predominantly from animal studies, that diets high in total fat, cholesterol, or trans fat may have an anxiogenic effect. With respect to a possible mechanism, there is evidence that inflammation plays an important role in the pathogenesis of psychiatric disorders [86], including anxiety [87], and that dietary fats can influence levels of inflammation [88]. Through their effects on enzyme pathways involved in the production of anti-inflammatory cytokines, omega-3 fatty acids contribute to lower levels of inflammation [89]. Conversely, omega-6 fatty acids increase levels of inflammation through increased pro-inflammatory cytokine production. Additionally, there is evidence that omega-3 fatty acids impact oxidative stress [90], neurotransmission [91], and neuroplasticity [92], which are known or hypothesized mechanisms for their use in the treatment of anxiety disorders [93,94]. Dietary omega-3 sources include fish and seafood, as well as flax seeds, chia and hemp seeds.

One somewhat inconsistent finding that has been observed in the present review is the impact of high fat diets. A large number of animal studies (39 of 63) reported a worsening of anxiety symptoms in response to intake of a high-fat diet. In contrast, the studies assessing the ketogenic diet, a diet that is very low in carbohydrates and generally high in fat content, suggest a possible therapeutic benefit. While these findings may be considered conflicting, it is speculated that the type of dietary fats used may have differed, with the high-fat diet delivered to the animals being composed of more omega-6, saturated and trans fatty acids. As such, the type of dietary fat, may be a significant factor in addition to the quantity of fat consumed.

### 4.5. Vitamins and Minerals

There is significant animal data suggesting an anxiolytic effect of several vitamins and minerals as well as supplemental formulas which deliver a combination or broad range of micronutrients. Given the presence of micronutrients in whole, unprocessed foods such as vegetables, fruit, and whole grains, these findings add evidence to the importance of eating a healthy diet containing a variety of unprocessed foods. Intake of foods that provide a rich source of zinc (oysters, crustaceans, meat, organ meat, leafy and root vegetables [95]), and selenium (Brazil nuts, seafood, meat, beans, and lentils [85]) could be prioritized.

Micronutrients such as zinc and selenium are necessary as coenzymes in the synthesis and regulation of neurotransmitters and neurotrophic factors [96] which may explain their importance in maintaining mental wellbeing. Additionally, B vitamins and folic acid contribute to the methylation balance which is hypothesized to be relevant to the pathophysiology of psychiatric illnesses [97].

### 4.6. Vegetables, Fruits, and Phytochemicals

There is fairly consistent evidence that vegetables, fruit, and plant constituents may exert anti-anxiety actions; however, the majority of the evidence comes from animal studies. Caffeine on its own or added to energy drinks appears to be associated with increased anxiety. Whole foods containing caffeine such as coffee, teas and cacao may have beneficial or equivocal impacts on anxiety, likely due to the co-occurrence of caffeine with other beneficial phytochemicals. Vegetables and fruit contribute to lower levels of inflammation and oxidative stress through their phytochemical and antioxidant constituents [98].

### 4.7. Food Allergy and Intolerance

The body of evidence related to the connection between food allergies and anxiety symptoms is limited and the majority of the evidence pertains to the presence of elevated anxiety symptoms among individuals with celiac disease and the anti-anxiety effects of implementing a gluten-free diet in this population. The presence of neuropsychiatric symptoms in celiac disease is established, with hypothesized mechanisms including micronutrient deficiency due to malabsorption and hyperhomocysteinemia [99]; however, it is unclear how the findings of these studies may apply to anxious individuals unaffected by celiac disease.

### 4.8. Gut Microbiome

Preliminary evidence suggests that the intake of beneficial microorganisms and prebiotic fiber may be beneficial in the treatment of anxiety. Habitual diet strongly influences the composition of the gut microbiome, thus adding more rationale for the inclusion of fruits, vegetables, fiber, and fermented foods in the diet [100]. Potential mechanisms for the impact of the microbiome on psychiatric wellbeing include the modulation of the production of gut peptides involved in the gut-brain axis [101] and neurotransmitter synthesis [102].

### 4.9. Strengths and Limitations

Strengths of the present review include an extremely rigorous search strategy intended to capture the full range of publications presenting data on this topic. A priori inclusion criteria and duplicate screening decreased the risk of bias. Completion of the project by an interdisciplinary team including clinicians and researchers contributed a range of perspectives and expertise.

The very large scope of this review was both a strength and limitation. Due to the very large volume of articles included in the review, in-depth analysis of individual articles was not possible. The results of the review may include over-simplification of the findings and a lack of attention to evaluating study quality, assessing study or publication bias, or providing contextual information (e.g., dose). In our data extraction and analysis we did not evaluate of the methods used for assessing participant anxiety symptoms or disorders, Anxiety symptoms can be assessed through a variety of methods including clinician- or self-administered questionnaires, or interviews which may utilize a range of diagnostic criteria. These different methods differ in their reliability as well as the exact nature of the symptoms or disorders that they assess. As a result, the studies included in this review report on relationships between food and a heterogeneous group of outcomes including the presence or absence of different anxiety disorders and a range of anxiety symptoms. The decision to include this heterogenous collection of research was an effort to capture a broad range of data related to this topic.

Another limitation of the present review is the unclear relevance of experimental studies which assessed the impact of high dose supplements of a dietary constituent. Doses of some of the nutrients delivered in trials as dietary supplement, such as zinc and omega-3 fatty acids, can be achieved through dietary modification; however, some of the nutrients, such as vitamin B6 (50 mg/day) were delivered in doses that cannot be achieved with food alone.

Another limitation was the exclusion of studies that failed to report changes in anxiety separately from other outcomes. Several studies that were not included in the present analysis reported ‘psychological distress’ as a composite of anxiety and depression symptoms but did not report anxiety results alone [103,104]. These studies were excluded from the present analysis as the purpose of this project was to identify research reporting anxiety outcomes specifically; however, it is noted that this resulted in the exclusion of a number of articles (*n* = 22).

The ability to draw conclusions from the data is also limited by a number of factors related to the methodology used in the included studies. This scoping review included a large number of animal studies which may have unclear applicability to humans. There are well established tests designed to measure changes in anxiety levels in animals through monitoring their behavior in a variety of experimental settings [105]; however, the applicability of these results to the human experience of anxiety is inherently limited. The benefits of animal research include the ability to manipulate dietary factors in a highly controlled environment, the ability to observe effects rapidly as a result of the animals’ reduced lifespan and the ability to withhold potentially beneficial nutrients. There were also a large number of observational studies, mostly cross-sectional in nature. This type of study cannot draw conclusions about causality. The association between diet and mental health is known to be highly complex and bidirectional. While there is robust evidence that dietary patterns impact the likelihood of developing mental illness [14], there is also evidence that mental illness impacts eating behaviors [106]. This occurs through changes in motivation and appetite that can results from mental illness [107] and metabolic changes, increased appetite and cravings, and gastrointestinal distress [108] that can occur as a results of psychiatric medications [109]. Additionally, confounding factors such as eating disorders may be responsible for associations that are present. Given this bidirectional relationship, the findings of cross-sectional studies have limited ability to answer the question of how food impacts anxiety. While a small number of prospective observational studies were identified in the present review, additional prospective studies are needed in order to accurately assess the impact of dietary patterns on the development of mental disorders, particularly the avoidance of potentially beneficial foods and increased intake of potentially harmful foods, which cannot be studied using an experimental design for ethical reasons.

Another important consideration when interpreting the study findings is the potential for difference between short- and long-term impacts of food on anxiety symptoms. As previously mentioned, it is known that the relationship between mental health symptoms and diet choices is bidirectional; emotional symptoms may drive eating behavior because of their immediate effects on the mitigation of emotional symptoms. The term “comfort eating” has been used to describe the phenomenon where individuals consume foods, especially those higher in calories, sugar, and fat, in response to negative affect [110]. Evidence from mechanistic studies suggest that corticosterone, a stress hormone, positively influences an animal’s intake of a sweet beverage [111] and that consumption of comfort food decreases mRNA production of hormones related to the stress response in animals [110]. It has been hypothesized that comfort eating is a behavior that decreases the stress-response during the experience of anxiety [110]. This phenomenon might explain some of the mixed finding of the present study. When considering the studies using “unhealthy”, “cafeteria”, or Western diets in animal models, 17 studies reported a decrease in anxiety symptoms and 21 reported an increase in anxiety symptoms. In contrast, of the 17 human observational studies assessing the relationship between unhealthy diet patterns and anxiety symptoms, 15 reported an association with more anxiety, and two reported no association. The mixed findings among the animal studies may be due to the duration of the experiment. Many of these studies assessed the impact of three to four weeks of the diet exposure on animal behavior and many of the studies reporting benefit assessed the impact of the diet on animals experiencing stress. The reported beneficial effects may be capturing the short-term stress-reducing effect of foods high in sugar and fat. In contrast, the human observational studies may have been capturing the effects of chronic consumption of unhealthy diets.

Another limitation that impacts the ability to draw clear conclusions from the present data is the enormous complexity of studying nutritional science. When considering the role of macronutrients (carbohydrates, protein, and fat), it is necessary to consider both the amount and type of the nutrient consumed. As highlighted previously, studies which categorized dietary patterns as high or low in macronutrients such as fat or carbohydrates may not have considered the types of fat or carbohydrates being consumed. Given the highly different health impacts of complex and refined carbohydrates, significant attention should be given to the studies differentiating these rather than those assessing total carbohydrates only.

Only a small number of intervention studies involved participants with anxiety disorders, many involved healthy participants or individuals with medical illnesses such as irritable bowel syndrome, diabetes, and cardiovascular disease. This has several implications. First, many of these studies were designed to assess cardiometabolic outcomes primarily and the studies may have not been adequately powered to detect changes in mental health symptoms. Secondly, the participants recruited to participate in these studies related to physical illness may have had low baseline levels of anxiety symptoms making it difficult to detect statistically significant changes in symptoms or becoming more susceptible to other scale attenuation effects (e.g., floor and ceiling effects). Thirdly, the impact of a nutritional intervention on a healthy or non-anxious individual may not be relevant to understanding how the intervention might impact individuals with clinically significant anxiety disorders. There is a clear need for intervention studies enrolling participants with anxiety disorders or elevated anxiety symptoms. Similarly, studies designed with changes in mental health symptoms as the primary outcomes are needed.

## 5. Conclusions

Although the results of this review reflect a field of study that is preliminary and emerging, the findings are consistent with established evidence about healthy eating patterns [112]. There is evidence of an association between healthy eating patterns and reduced anxiety symptoms. In the absence of a contraindication such as an allergy or specific medical condition, dietary interventions are considered low in risk, cost-effective [113], may confer secondary benefit to physical aspects of health and have at least some evidence suggesting a beneficial effect. However, the delivery of nutritional counselling as part of the treatment of anxiety disorders by primary care, psychiatry, dieticians, naturopaths, or other care providers is currently limited.

Prospective observational studies are needed to more clearly establish the causal role of diet factors in the development and progression of anxiety disorders. High quality intervention studies involving participants with anxiety disorders are warranted in order to evaluate the therapeutic potential of nutrition interventions in the management of anxiety disorders.

## Figures and Tables

**Table 1:** Summary of nutrients and diet patterns associated with more or less anxiety symptom severity or disorder prevalence.

Association with Less Anxiety	Association with More Anxiety
Vegetables and Fruit Omega-3 Fatty Acids, Alpha-lipoic acid, Omega-9 Fatty acids Nuts and seeds “Healthy” Dietary Patterns, Mediterranean diet, Traditional Dietary Patterns, Anti-inflammatory diet pattern Caloric Restriction Fasting or intermittent fasting Breakfast Consumption Broad-Spectrum Micronutrients Vegan Diet Zinc, Magnesium, Selenium Vitamin C, Vitamin E, Choline Ketogenic Diet Food sources of *Lactobacillus* and *Bifidobacterium* Culinary herbs, Turmeric, Saffron, Soy, Green tea, Herbal tea, Quercetin, Resveratrol, other phytochemicals (flavonoids, polyphenols, carotenoids)	High-fat diet, high cholesterol, high trans fat Inadequate tryptophan and dietary protein High intake of sugar and refined carbohydrates, artificial sweeteners “Unhealthy” Dietary Patterns, typically defined as high in unhealthy fats and refined sugars Snacking

## Data Availability

Supporting data available on request.

## Supplementary Materials

The following are available online at https://www.mdpi.com/article/10.3390/nu13124418/s1, File S1: Search Strategy, File S2: Excluded studies, File S3: All included studies.

## References

[1] Gale C.K., Millichamp J. (2011). Generalised anxiety disorder. BMJ Clin. Evid..

[2] Kim Y.-K. (2019). Panic Disorder: Current Research and Management Approaches. Psychiatry Investig..

[3] Wittchen H.-U., Gloster A.T., Beesdo-Baum K., Fava G.A., Craske M.G. (2010). Agoraphobia: A review of the diagnostic classificatory position and criteria. Depress. Anxiety.

[4] Glue P., Canton J., Scott K.M. (2012). Optimal treatment of social phobia: Systematic review and meta-analysis. Neuropsychiatr. Dis. Treat..

[5] Eaton W.W., Bienvenu O.J., Miloyan B. (2018). Specific phobias. Lancet Psychiatry.

[6] Louise P., Siobhan O., Louise M., Jean G., Pelletier L., O’Donnell S., McRae L., Grenier J. (2017). The burden of generalized anxiety disorder in Canada. Health Promot. Chronic Dis. Prev. Can..

[7] Saarni S.I., Suvisaari J., Sintonen H., Pirkola S., Koskinen S., Aromaa A., Lönnqvist J. (2007). Impact of psychiatric disorders on health-related quality of life: General population survey. Br. J. Psychiatry.

[8] Revicki D.A., Travers K., Wyrwich K.W., Svedsäter H., Locklear J., Mattera M.S., Sheehan D.V., Montgomery S. (2012). Humanistic and economic burden of generalized anxiety disorder in North America and Europe. J. Affect. Disord..

[9] Kessler R.C., McGonagle K.A., Zhao S., Nelson C.B., Hughes M., Eshleman S., Wittchen H.-U., Kendler K.S. (1994). Lifetime and 12-Month Prevalence of DSM-III-R Psychiatric Disorders in the United States. Arch. Gen. Psychiatry.

[10] Gliatto M.F. (2000). Generalized anxiety disorder. Am. Fam. Physician.

[11] Collins K.A., Westra H.A., Dozois D.J., Burns D.D. (2004). Gaps in accessing treatment for anxiety and depression: Challenges for the delivery of care. Clin. Psychol. Rev..

[12] Jacka F.N., O’Neil A., Opie R., Itsiopoulos C., Cotton S., Mohebbi M., Castle D., Dash S., Mihalopoulos C., Chatterton M.L. (2017). A randomised controlled trial of dietary improvement for adults with major depression (the ‘SMILES’ trial). BMC Med..

[13] Parletta N., Zarnowiecki D., Cho J., Wilson A., Bogomolova S., Villani A., Itsiopoulos C., Niyonsenga T., Blunden S., Meyer B. (2019). A Mediterranean-style dietary intervention supplemented with fish oil improves diet quality and mental health in people with depression: A randomized controlled trial (HELFIMED). Nutr. Neurosci..

[14] Marx W., Moseley G., Berk M., Jacka F. (2017). Nutritional psychiatry: The present state of the evidence. Proc. Nutr. Soc..

[15] Xu Y., Zeng L., Zou K., Shan S., Wang X., Xiong J., Zhao L., Zhang L., Cheng G. (2021). Role of dietary factors in the prevention and treatment for depression: An umbrella review of meta-analyses of prospective studies. Transl. Psychiatry.

[16] Arksey H., O’Malley L. (2005). Scoping studies: Towards a methodological framework. Int. J. Soc. Res. Methodol..

[17] Aucoin M., LaChance L., Cooley K., Kidd S. (2020). Diet and Psychosis: A Scoping Review. Neuropsychobiology.

[18] Gates A., Johnson C., Hartling L. (2018). Technology-assisted title and abstract screening for systematic reviews: A retrospective evaluation of the Abstrackr machine learning tool. Syst. Rev..

[19] Rathbone J., Hoffmann T., Glasziou P. (2015). Faster title and abstract screening? Evaluating Abstrackr, a semi-automated online screening program for systematic reviewers. Syst. Rev..

[20] Gates A., Gates M., Sebastianski M., Guitard S., Elliott S.A., Hartling L. (2020). The semi-automation of title and abstract screening: A retrospective exploration of ways to leverage Abstrackr’s relevance predictions in systematic and rapid reviews. BMC Med. Res. Methodol..

[21] Clappison E., Hadjivassiliou M., Zis P. (2020). Psychiatric Manifestations of Coeliac Disease, a Systematic Review and Meta-Analysis. Nutrients.

[22] Hunsche C., de Toda I.M., De la Fuente M. (2019). Impacts of the late adulthood diet-induced obesity onset on behavior, immune function, redox state and life span of male and female mice. Brain Behav. Immun..

[23] Souza C., Moreira J.D., Siqueira I., Pereira A., Rieger D., Souza D., Souza T., Portela L., Perry M. (2007). Highly palatable diet consumption increases protein oxidation in rat frontal cortex and anxiety-like behavior. Life Sci..

[24] Clemente-Suárez V.J. (2020). Multidisciplinary intervention in the treatment of mixed anxiety and depression disorder. Physiol. Behav..

[25] Null G., Pennesi L., Feldman M. (2017). Nutrition and Lifestyle Intervention on Mood and Neurological Disorders. J. Evid.-Based Integr. Med..

[26] Cooley K., Szczurko O., Perri D., Mills E.J., Bernhardt B., Zhou Q., Seely D. (2009). Naturopathic Care for Anxiety: A Randomized Controlled Trial ISRCTN78958974. PLoS ONE.

[27] Forsyth A., Deane F.P., Williams P. (2015). A lifestyle intervention for primary care patients with depression and anxiety: A randomised controlled trial. Psychiatry Res..

[28] Laporte D.J. (1992). Treatment response in obese binge eaters: Preliminary results using a very low calorie diet (VLCD) and behavior therapy. Addict. Behav..

[29] Ein N., Armstrong B., Vickers K. (2018). The effect of a very low calorie diet on subjective depressive symptoms and anxiety: Meta-analysis and systematic review. Int. J. Obes..

[30] Jebeile H., Gow M.L., Baur L.A., Garnett S.P., Paxton S.J., Lister N.B. (2019). Association of Pediatric Obesity Treatment, Including a Dietary Component, with Change in Depression and Anxiety: A Systematic Review and Meta-analysis. JAMA Pediatr..

[31] Hillman L.C., Stace N.H., Pomare E.W. (1984). Irritable bowel patients and their long-term response to a high fiber diet. Am. J. Gastroenterol..

[32] Christensen L., Krietsch K., White B., Stagner B. (1985). Impact of a dietary change on emotional distress. J. Abnorm. Psychol..

[33] Mohammed H., Ghosh S., Vuvor F., Mensah-Armah S., Steiner-Asiedu M. (2016). Dietary intake and the dynamics of stress, hypertension and obesity in a peri-urban community in Accra. Ghana Med. J..

[34] Mesgarani M., Hosseinbor M., Shafiee S., Sarkoubi R. (2016). The Relationship of Parental Mental Health and Dietary Pattern with Adolescent Mental Health. Int. J. High Risk Behav. Addict..

[35] Hudson C., Hudson S., MacKenzie J. (2007). Protein-source tryptophan as an efficacious treatment for social anxiety disorder: A pilot studyThis article is one of a selection of papers published in this special issue (part 1 of 2) on the Safety and Efficacy of Natural Health Products. Can. J. Physiol. Pharmacol..

[36] Capello A.E., Markus C.R. (2014). Effect of sub chronic tryptophan supplementation on stress-induced cortisol and appetite in subjects differing in 5-HTTLPR genotype and trait neuroticism. Psychoneuroendocrinology.

[37] Smith C.A., Shewamene Z., Galbally M., Schmied V., Dahlen H. (2019). The effect of complementary medicines and therapies on maternal anxiety and depression in pregnancy: A systematic review and meta-analysis. J. Affect. Disord..

[38] Deane K.H.O., Jimoh O.F., Biswas P., O’Brien A., Hanson S., Abdelhamid A.S., Fox C., Hooper L. (2021). Omega-3 and polyunsaturated fat for prevention of depression and anxiety symptoms: Systematic review and meta-analysis of randomised trials. Br. J. Psychiatry.

[39] Su K.-P., Tseng P.-T., Lin P.-Y., Okubo R., Chen T.-Y., Chen Y.-W., Matsuoka Y.J. (2018). Association of Use of Omega-3 Polyunsaturated Fatty Acids with Changes in Severity of Anxiety Symptoms: A Systematic Review and Meta-analysis. JAMA Netw. Open.

[40] Yehuda S., Rabinovitz S., Mostofsky D.I. (2005). Mixture of essential fatty acids lowers test anxiety. Nutr. Neurosci..

[41] Abdul-Razzak K.K., Almanasrah S.O., Obeidat B.A., Khasawneh A.G. (2018). Vitamin D is a potential antidepressant in psychiatric outpatients. Int. J. Clin. Pharmacol. Ther..

[42] Rucklidge J.J., Andridge R., Gorman B., Blampied N., Gordon H., Boggis A. (2012). Shaken but unstirred? Effects of micronutrients on stress and trauma after an earthquake: RCT evidence comparing formulas and doses. Hum. Psychopharmacol. Clin. Exp..

[43] Gautam M., Agrawal M., Gautam M., Sharma P., Gautam A.S., Gautam S. (2012). Role of antioxidants in generalised anxiety disorder and depression. Indian J. Psychiatry.

[44] Jaatinen N., Korpela R., Poussa T., Turpeinen A., Mustonen S., Merilahti J., Peuhkuri K. (2013). Effects of daily intake of yoghurt enriched with bioactive components on chronic stress responses: A double-blinded randomized controlled trial. Int. J. Food Sci. Nutr..

[45] Russo A. (2011). Decreased Zinc and Increased Copper in Individuals with Anxiety. Nutr. Metab. Insights.

[46] Choi E.B., Lee J.E., Hwang J.-Y. (2018). Fruit and vegetable intakes in relation to behavioral outcomes associated with a nutrition education intervention in preschoolers. Nutr. Res. Pract..

[47] Conner T.S., Brookie K.L., Carr A.C., Mainvil L.A., Vissers M.C.M. (2017). Let them eat fruit! The effect of fruit and vegetable consumption on psychological well-being in young adults: A randomized controlled trial. PLoS ONE.

[48] Smith A.P., Rogers R. (2014). Positive Effects of a Healthy Snack (Fruit) Versus an Unhealthy Snack (Chocolate/Crisps) on Subjective Reports of Mental and Physical Health: A Preliminary Intervention Study. Front. Nutr..

[49] Camfield D., Stough C., Farrimond J., Scholey A.B. (2014). Acute effects of tea constituents L-theanine, caffeine, and epigallocatechin gallate on cognitive function and mood: A systematic review and meta-analysis. Nutr. Rev..

[50] Hieu T.H., Dibas M., Dila K.A.S., Sherif N.A., Hashmi M.U., Mahmoud M., Trang N.T.T., Abdullah L., Nghia T.L.B., Mai Nhu Y. (2019). Therapeutic efficacy and safety of chamomile for state anxiety, generalized anxiety disorder, insomnia, and sleep quality: A systematic review and meta-analysis of randomized trials and quasi-randomized trials. Phytotherapy Res..

[51] Marx W., Lane M., Rocks T., Ruusunen A., Loughman A., Lopresti A., Marshall S., Berk M., Jacka F., Dean O.M. (2019). Effect of saffron supplementation on symptoms of depression and anxiety: A systematic review and meta-analysis. Nutr. Rev..

[52] Fusar-Poli L., Vozza L., Gabbiadini A., Vanella A., Concas I., Tinacci S., Petralia A., Signorelli M., Aguglia E. (2020). Curcumin for depression: A meta-analysis. Crit. Rev. Food Sci. Nutr..

[53] Ng Q.X., Koh S.S.H., Chan H.W., Ho C.Y.X. (2017). Clinical Use of Curcumin in Depression: A Meta-Analysis. J. Am. Med. Dir. Assoc..

[54] Farzaei M.H., Rahimi R., Nikfar S., Abdollahi M. (2018). Effect of resveratrol on cognitive and memory performance and mood: A meta-analysis of 225 patients. Pharmacol. Res..

[55] Keefe J.R., Guo W., Li Q.S., Amsterdam J.D., Mao J.J. (2018). An exploratory study of salivary cortisol changes during chamomile extract therapy of moderate to severe generalized anxiety disorder. J. Psychiatr. Res..

[56] Amsterdam J.D., Li Y., Soeller I., Rockwell K., Mao J.J., Shults J. (2009). A Randomized, Double-Blind, Placebo-Controlled Trial of Oral Matricaria recutita (Chamomile) Extract Therapy for Generalized Anxiety Disorder. J. Clin. Psychopharmacol..

[57] Mao J.J., Xie S.X., Keefe J.R., Soeller I., Li Q.S., Amsterdam J.D. (2016). Long-term chamomile (*Matricaria chamomilla* L.) treatment for generalized anxiety disorder: A randomized clinical trial. Phytomedicine.

[58] Jafarnia N., Ghorbani Z., Nokhostin M., Manayi A., Nourimajd S., Jahromi S.R. (2017). Effect of Saffron (*Crocus satious* L.) as an Add-On Therapy to Sertraline in Mild to Moderate Generalized Anxiety Disorder: A Double Blind Randomized Controlled Trial. Arch. Neurosci..

[59] Milajerdi A., Jazayeri S., Shirzadi E., Hashemzadeh N., Azizgol A., Djazayery A., Esmaillzadeh A., Akhondzadeh S. (2018). The effects of alcoholic extract of saffron (*Crocus satious* L.) on mild to moderate comorbid depression-anxiety, sleep quality, and life satisfaction in type 2 diabetes mellitus: A double-blind, randomized and placebo-controlled clinical trial. Complement. Ther. Med..

[60] Sudheeran S.P., Jacob D., Mulakal J.N., Nair G.G., Maliakel A., Maliakel B., Ramadasan K., Krishnakumar I.M. (2016). Safety, Tolerance, and Enhanced Efficacy of a Bioavailable Formulation of Curcumin with Fenugreek Dietary Fiber on Occupational Stress: A Randomized, Double-Blind, Placebo-Controlled Pilot Study. J. Clin. Psychopharmacol..

[61] Sarris J., Byrne G.J., Cribb L., Oliver G., Murphy J., Macdonald P., Nazareth S., Karamacoska D., Galea S., Short A. (2019). L-theanine in the adjunctive treatment of generalized anxiety disorder: A double-blind, randomised, placebo-controlled trial. J. Psychiatr. Res..

[62] Pinto-Sanchez M.I., Hall G.B., Ghajar K., Nardelli A., Bolino C., Lau J.T., Martin F.-P., Cominetti O., Welsh C., Rieder A. (2017). Probiotic Bifidobacterium longum NCC3001 Reduces Depression Scores and Alters Brain Activity: A Pilot Study in Patients with Irritable Bowel Syndrome. Gastroenterology.

[63] Eskandarzadeh S., Effatpanah M., Khosravi-Darani K., Askari R., Hosseini A.F., Reisian M., Jazayeri S. (2021). Efficacy of a multispecies probiotic as adjunctive therapy in generalized anxiety disorder: A double blind, randomized, placebo-controlled trial. Nutr. Neurosci..

[64] Liu B., He Y., Wang M., Liu J., Ju Y., Zhang Y., Liu T., Li L., Li Q. (2018). Efficacy of probiotics on anxiety-A meta-analysis of randomized controlled trials. Depress. Anxiety.

[65] Liu R.T., Walsh R.F.L., Sheehan A.E. (2019). Prebiotics and probiotics for depression and anxiety: A systematic review and meta-analysis of controlled clinical trials. Neurosci. Biobehav. Rev..

[66] Reis D.J., Ilardi S.S., Punt S.E.W. (2018). The anxiolytic effect of probiotics: A systematic review and meta-analysis of the clinical and preclinical literature. PLoS ONE.

[67] Del Olmo N., Blanco-Gandia M.C., Mateos-Garcia A., Del Rio D., Minarro J., Ruiz-Gayo M., Rodríguez-Arias M. (2019). Differential Impact of Ad Libitum or Intermittent High-Fat Diets on Bingeing Ethanol-Mediated Behaviors. Nutrients.

[68] Dornellas A.P.S., Boldarine V.T., Pedroso A.P., Carvalho L.O.T., de Andrade I.S., Vulcani-Freitas T.M., dos Santos C.C.C., da Penha Oller do Nascimento C.M., Oyama L.M., Ribeiro E.B. (2018). High-Fat Feeding Improves Anxiety-Type Behavior Induced by Ovariectomy in Rats. Front. Neurosci..

[69] Segat H., Barcelos R., Metz V., Rosa H., Roversi K., Antoniazzi C., Vey L., Kronbauer M., Veit J., Piccolo J. (2017). Influence of physical activity on addiction parameters of rats exposed to amphetamine which were previously supplemented with hydrogenated vegetable fat. Brain Res. Bull..

[70] Segev Y., Livne A., Mints M., Rosenblum K. (2016). Concurrence of High Fat Diet and APOE Gene Induces Allele Specific Metabolic and Mental Stress Changes in a Mouse Model of Alzheimer’s Disease. Front. Behav. Neurosci..

[71] Otsuka A., Shiuchi T., Chikahisa S., Shimizu N., Séi H. (2019). Sufficient intake of high-fat food attenuates stress-induced social avoidance behavior. Life Sci..

[72] Haleem D.J., Mahmood K. (2021). Brain serotonin in high-fat diet-induced weight gain, anxiety and spatial memory in rats. Nutr. Neurosci..

[73] Hu F.B., Manson J.E., Willett W.C. (2001). Types of Dietary Fat and Risk of Coronary Heart Disease: A Critical Review. J. Am. Coll. Nutr..

[74] Ramsden C.E., Zamora D., Majchrzak-Hong S., Faurot K., Broste S.K., Frantz R., Davis J.M., Ringel A., Suchindran C.M., Hibbeln J.R. (2016). Re-evaluation of the traditional diet-heart hypothesis: Analysis of recovered data from Minnesota Coronary Experiment (1968–73). BMJ.

[75] Sergentanis T., Chelmi M.-E., Liampas A., Yfanti C.-M., Panagouli E., Vlachopapadopoulou E., Michalacos S., Bacopoulou F., Psaltopoulou T., Tsitsika A. (2020). Vegetarian Diets and Eating Disorders in Adolescents and Young Adults: A Systematic Review. Children.

[76] Swinbourne J.M., Touyz S. (2007). The co-morbidity of eating disorders and anxiety disorders: A review. Eur. Eat. Disord. Rev..

[77] Woo K.S., Kwok T.C., Celermajer D.S. (2014). Vegan Diet, Subnormal Vitamin B-12 Status and Cardiovascular Health. Nutrients.

[78] Lakin V., Haggarty P., Abramovich D., Ashton J., Moffat C., McNeill G., Danielian P., Grubb D. (1998). Dietary intake and tissue concentration of fatty acids in omnivore, vegetarian and diabetic pregnancy. Prostaglandins Leukot. Essent. Fat. Acids.

[79] Anderson R.J., Grigsby A.B., Freedland K.E., De Groot M., McGill J.B., Clouse R.E., Lustman P.J. (2002). Anxiety and poor glycemic control: A meta-analytic review of the literature. Int. J. Psychiatry Med..

[80] Moja E.A., Cipolla P., Castoldi D., Tofanetti O. (1989). Dose-response decrease in plasma tryptophan and in brain tryptophan and serotonin after tryptophan-free amino acid mixtures in rats. Life Sci..

[81] Stein D.J., Stahl S. (2000). Serotonin and anxiety: Current models. Int. Clin. Psychopharmacol..

[82] Cynober L., Bier D.M., Kadowaki M., Morris J.S.M., Elango R., Smriga M. (2016). Proposals for Upper Limits of Safe Intake for Arginine and Tryptophan in Young Adults and an Upper Limit of Safe Intake for Leucine in the Elderly. J. Nutr..

[83] Seltzer S., Dewart D., Pollack R.L., Jackson E. (1982). The effects of dietary tryptophan on chronic maxillofacial pain and experimental pain tolerance. J. Psychiatr. Res..

[84] Alusik S., Kalatova D., Paluch Z. (2014). Serotonin syndrome. Neuroendocrinol. Lett..

[85] U.S. Department of Agriculture, Agricultural Research Service (2019). FoodData Central. Fdc.nal.usda.gov.

[86] Firth J., Veronese N., Cotter J., Shivappa N., Hebert J.R., Ee C., Smith L., Stubbs B., Jackson S.E., Sarris J. (2019). What Is the Role of Dietary Inflammation in Severe Mental Illness? A Review of Observational and Experimental Findings. Front. Psychiatry.

[87] Salim S., Chugh G., Asghar M. (2012). Inflammation in Anxiety. Adv. Protein Chem. Struct. Biol..

[88] Calder P.C. (2006). Polyunsaturated fatty acids and inflammation. Prostaglandins Leukot. Essent. Fat. Acids.

[89] Bäck M., Hansson G.K. (2019). Omega-3 fatty acids, cardiovascular risk, and the resolution of inflammation. FASEB J..

[90] Chen J., Wang D., Zong Y., Yang X. (2021). DHA Protects Hepatocytes from Oxidative Injury through GPR120/ERK-Mediated Mitophagy. Int. J. Mol. Sci..

[91] DeLion S., Chalon S., Hérault J., Guilloteau D., Besnard J.C., Durand G. (1994). Chronic dietary alpha-linolenic acid deficiency alters dopaminergic and serotoninergic neurotransmission in rats. J. Nutr..

[92] Marino A., Cuzzocrea S. (2013). n-3 Fatty Acids: Role in Neurogenesis and Neuroplasticity. Curr. Med. Chem..

[93] Gałecki P., Mossakowska-Wójcik J., Talarowska M. (2018). The anti-inflammatory mechanism of antidepressants—SSRIs, SNRIs. Prog. Neuro-Psychopharmacol. Biol. Psychiatry.

[94] Branchi I., Santarelli S., Capoccia S., Poggini S., D’Andrea I., Cirulli F., Alleva E. (2013). Antidepressant Treatment Outcome Depends on the Quality of the Living Environment: A Pre-Clinical Investigation in Mice. PLoS ONE.

[95] Solomons N.W. (2001). Dietary Sources of Zinc and Factors Affecting its Bioavailability. Food Nutr. Bull..

[96] Rucklidge J.J., Kaplan B.J. (2013). Broad-spectrum micronutrient formulas for the treatment of psychiatric symptoms: A systematic review. Expert Rev. Neurother..

[97] Abdolmaleky H.M., Smith C., Faraone S., Shafa R., Stone W., Glatt S., Tsuang M.T. (2004). Methylomics in psychiatry: Modulation of gene-environment interactions may be through DNA methylation. Am. J. Med. Genet..

[98] Zhu F., Du B., Xu B. (2018). Anti-inflammatory effects of phytochemicals from fruits, vegetables, and food legumes: A review. Crit. Rev. Food Sci. Nutr..

[99] Trovato C.M., Raucci U., Valitutti F., Montuori M., Villa M.P., Cucchiara S., Parisi P. (2019). Neuropsychiatric manifestations in celiac disease. Epilepsy Behav..

[100] Wastyk H.C., Fragiadakis G.K., Perelman D., Dahan D., Merrill B.D., Yu F.B., Topf M., Gonzalez C.G., Van Treuren W., Han S. (2021). Gut-microbiota-targeted diets modulate human immune status. Cell.

[101] Lach G., Schellekens H., Dinan T.G., Cryan J.F. (2018). Anxiety, Depression, and the Microbiome: A Role for Gut Peptides. Neurotherapeutics.

[102] Shafiq J., Khan B., Saleem F., Abbas G., Ahmed A. (2020). Gut microbiome interferes with host tryptophan metabolism pathway and regulate basal anxiety–like behavior. Int. J. Infect. Dis..

[103] Arbour-Nicitopoulos K.P., Faulkner G., Irving H.M. (2012). Multiple Health-Risk Behaviour and Psychological Distress in Adolescence. J. Can. Acad. Child Adolesc. Psychiatry.

[104] Grønning K., Espnes G.A., Nguyen C., Rodrigues A.M.F., Gregorio M.J., Sousa R., Canhão H., André B. (2018). Psychological distress in elderly people is associated with diet, wellbeing, health status, social support and physical functioning—A HUNT3 study. BMC Geriatr..

[105] Steimer T. (2011). Animal models of anxiety disorders in rats and mice: Some conceptual issues. Dialog. Clin. Neurosci..

[106] van der Pols J.C. (2018). Nutrition and mental health: Bidirectional associations and multidimensional measures. Public Health Nutr..

[107] Macht M., Roth S., Ellgring H. (2002). Chocolate eating in healthy men during experimentally induced sadness and joy. Appetite.

[108] Willner P., Benton D., Brown E., Cheeta S., Roderique-Davies G., Morgan J., Morgan M. (1998). “Depression” increases “craving” for sweet rewards in animal and human models of depression and craving. Psychopharmacology.

[109] Shobo M., Yamada H., Mihara T., Kondo Y., Irie M., Harada K., Ni K., Matsuoka N., Kayama Y. (2011). Two models for weight gain and hyperphagia as side effects of atypical antipsychotics in male rats: Validation with olanzapine and ziprasidone. Behav. Brain Res..

[110] Dallman M.F., Pecoraro N., Akana S.F., la Fleur S.E., Gomez F., Houshyar H., Bell M.E., Bhatnagar S., Laugero K.D., Manalo S. (2003). Chronic stress and obesity: A new view of “comfort food”. Proc. Natl. Acad. Sci. USA.

[111] Bhatnagar S., Bell M.E., Liang J., Soriano L., Nagy T.R., Dallman M.F. (2000). Corticosterone Facilitates Saccharin Intake in Adrenalectomized Rats: Does Corticosterone Increase Stimulus Salience?. J. Neuroendocr..

[112] (2007). Health Canada, Eating Well with Canada’s Food Guide. http://www.hc-sc.gc.ca/fn-an/food-guide-aliment/index-eng.php.

[113] Chatterton M.L., Mihalopoulos C., O’Neil A., Itsiopoulos C., Opie R., Castle D., Dash S., Brazionis L., Berk M., Jacka F. (2018). Economic evaluation of a dietary intervention for adults with major depression (the “SMILES” trial). BMC Public Health.

