# Supplementary material for: Diet and Anxiety: A Scoping Review
Source: Nutrients. 2021 Dec 10;13(12):4418. doi: 10.3390/nu13124418 (PMC8706568; doi:10.3390/nu13124418)
Supplement: Supplementary file 1 [file nutrients-13-04418-s001.zip › nutrients-1410594-supplementary/Supplemental File S1 Search Strategy.pdf]

Database: Ovid MEDLINE: Epub Ahead of Print, In-Process & Other Non-Indexed Citations, Ovid MEDLINE® Daily and Ovid MEDLINE® <1946-Present>
Search Strategy:
----
1 exp Anxiety Disorders/ (78375)
2 exp Anxiety/ (82991)
3 (anxiety or anxieties or anxious*).tw,kf. (195409)
4 (hypervigilan* or hyper-vigilan* or nervousness).tw,kf. (2486)
5 ((incessan* or obsessive* or persistan* or intense*) adj3 fear*).tw,kf. (406)
6 (agoraphobi* or claustrophobi*).tw,kf. (3862)
7 (neurocirculatory asthenia* or cardiac neuros#s or effort syndrome* or hyperkinetic heart syndrome*).tw,kf. (664)
8 (neurotic disorder* or neuros#s or psychoneuros#s or psycho-neuros#s).tw,kf. (11962)
9 (obsessive-compulsive or anankastic personalit* or compulsive personalit* or obsessive personalit* or hoarding or psychastheni*).tw,kf. (18854)
10 (panic disorder* or (panic adj2 attack*)).tw,kf. (11172)
11 (phobia or phobias or phobic).tw,kf. (11549)
12 (catastrophi#ation? or catastrophi#e* or catastrophi#ing).tw,kf. (3390)
13 or/1-12 (273657)
14 exp Diet Therapy/ (53629)
15 exp Nutritional Support/ (44653)
16 exp Anxiety Disorders/dh (32)
17 Anxiety/dh (67)
18 Beverages/ or exp Carbonated Beverages/ or Coffee/ or exp Drinking Water/ or exp Energy Drinks/ or "Fruit and Vegetable Juices"/ or exp Milk/ or exp Tea/ or exp Teas, Herbal/ (121024)
19 exp Food/ (1266550)
20 (bread* or candy or candies or condiment$1 or dairy produc* or egg or eggs or flour or flours or fruit or fruits or honey or meal or meals or breakfast* or lunch$2 or dinner$1 or supper$1 or meat or meats or poultry or seafood or shellfish* or fish or fishes or molasses or nut or nuts or seed or seeds or grain or grains or vegetable$1).tw,kf. (719715)
21 (chicken or turkey or beef or pork or ham or bacon).tw,kf. (145494)
22 Salmon/ or (oncorhynchus or salmo salar or salmon?).tw,kf. (22189)
23 Pectinidae/ or (pectinidae or scallop?).tw,kf. (2295)
24 Penaeidae/ or (penaeidae or shrimp?).tw,kf. (11992)
25 Tuna/ or tuna.tw,kf. (2741)
26 Bivalvia/ or (clam or clams or mussel?).tw,kf. (16469)
27 Ostrea/ or (ostrea or oyster?).tw,kf. (7303)
28 Perciformes/ or (mackerel? or sardine?).tw,kf. (9615)
29 Chenopodium quinoa/ or quinoa?.tw,kf. (992)
30 Millets/ or millet?.tw,kf. (2510)
31 Fagopyrum/ or (fagopyrum or buckwheat?).tw,kf. (1535)
32 exp Edible Grain/ or cereal?.tw,kf. (31811)
33 Daucus carota/ or (daucus or carrot?).tw,kf. (5669)
34 exp Brassica/ or (brassica or broccoli? or brussel sprout? or cabbage? or cauliflower? or collard green? or kale or rape seed? or rapeseed? or rutabaga? or turnip?).tw,kf. (26629)
35 Zea mays/ or (zea mays or corn?).tw,kf. (59675)
36 Onions/ or (allium cepa or allium porrum or leek? or onion?).tw,kf. (7795)
37 Cucumis sativus/ or (cucumis sativus or cucumber?).tw,kf. (8578)

38 Persea/ or (persea or avocado?).tw,kf. (1556)
39 Lettuce/ or (lettuce? or lactuca sativa).tw,kf. (6013)
40 Cucurbita/ or (cucurbita or squash or squashes or pumpkin?).tw,kf. (4477)
41 Solanum tuberosum/ or (solanum tuberosum or potato*).tw,kf. (26435)
42 Malus/ or (malus or apple? or crabapple?).tw,kf. (16736)
43 Musa/ or (musa or banana?).tw,kf. (5417)
44 exp Cucumis/ or (cucumis or cantaloupe? or melon or melons).tw,kf. (6437)
45 Actinidia/ or (actinidia or kiwi or kiwis).tw,kf. (1659)
46 Pyrus/ or (pyrus or pear?).tw,kf. (9923)
47 exp Prunus/ or (plum or plums or prunus).tw,kf. (7043)
48 Ananas/ or (ananas or pineapple?).tw,kf. (1612)
49 Yogurt/ or (yogurt? or yoghurt?).tw,kf. (4586)
50 Cheese/ or (cheese? or curd or curds).tw,kf. (12961)
51 Lens Plant/ or (lens culinaris or lens plant? or lentil?).tw,kf. (2919)
52 pulses.tw,kf. (58228)
53 Cicer/ or (Cicer or chickpea? or chick pea? or garbanzo?).tw,kf. (2476)
54 Arachis/ or (arachis or peanut?).tw,kf. (14656)
55 Prunus dulcis/ or (prunus dulcis or prunis communis or amygdalus communis or almond?).tw,kf. (2777)
56 Juglans/ or (juglans or walnut?).tw,kf. (2661)
57 Pistacia/ or (pistacia or pistachio?).tw,kf. (1377)
58 Anacardium/ or (anacardium or cashew?).tw,kf. (1168)
59 Chia.tw,kf. (918)
60 Rapeseed Oil/ or (rapeseed oil? or canola? or LEAR oil?).tw,kf. (4379)
61 (jam or jams).tw,kf. (2038)
62 Soybeans/ or (Glycine max or soybean? or soy or soya).tw,kf. (58925)
63 (bean curd? or miso? or natto? or tempeh? or tofu?).tw,kf. (1900)
64 (pasta? or spaghetti? or linguine? or ravioli?).tw,kf. (2531)
65 (noodle or noodles or pizza?).tw,kf. (1541)
66 (stirfry* or stir-fry* or stirfried or stir-fried).tw,kf. (220)
67 (hamburger? or hotdog? or hot dog? or chili or soup or soups).tw,kf. (5701)
68 (French toast* or pancake? or waffle? or porridge? or omelet*).tw,kf. (1587)
69 sausage?.tw,kf. (4111)
70 salad?.tw,kf. (2242)
71 (cake or cakes or cookie? or dessert? or pie or pies or muffin?).tw,kf. (10213)
72 (chip or chips or dorito* or popcorn or pretzel* or snack* or taco or tacos).tw,kf. (63318)
73 exp Nutritional Physiological Phenomena/ (585511)
74 (glycemic index* or glycemia load$1).tw,kf. (2384)
75 (diet or diets or dietary).tw,kf,hw. (647741)
76 (eat or eats or eating).tw,kf. (88068)
77 (food or foods or feed or feeds or feeding or fed).tw,kf,hw. (989945)
78 (nutrition* or nutritive* or nutrient* or macronutrient* or macro-nutrient* or micronutrient* or micro-nutrient*).tw,kf,hw. (502603)
79 (vegan or vegans or veganism or vegetarianism or macro?biotic*).tw,kf. (1635)
80 (whole food? or "plant-based" or WFPB).tw,kf. (5038)
81 ((egg$ or milk$1 or whey$1 or vegetable$1) adj2 protein$1).tw,kf. (17236)
82 (butter$1 or ghee or margarine$1 or cholesterol* or unsaturated fat$1 or unsaturated oil$1 or saturated fat$1 or saturated oil$1 or trans fat$1 or transfat* or trans oil$1 or polyunsaturated fat$1 or

polyunsaturated oil$1 or poly-unsaturated fat$1 or poly-unsaturated oil$1 or monounsaturated fat$1 or monounsaturated oil$1 or mono-unsaturated fat$1 or mono-unsaturated oil$1).tw,kf. (253003)
83 (fiber or fibre or roughage or wheat bran$1).tw,kf. (191425)
84 (carbohydrate* or sugar* or starch* or sucrose* or syrup*).tw,kf. (334392)
85 (glucose* or fructose* or isoglucose*).tw,kf. (480824)
86 exp Food Hypersensitivity/ (19971)
87 ((food or egg or eggs or milk or nut or nuts or peanut or peanuts or shellfish* or wheat) adj3 (allerg* or hypersensitiv* or react* or sensitiv*)).tw,kf. (22714)
88 (beverage? or drink or drinks or drinking or drank or drunk or juice? or soda or sodas or pop or pops or refreshment? or tea or teas or chai or coffee? or punch$2 or milk? or water or waters).tw,kf. (1070334)
89 coke.tw. not Coke/ (1292)
90 (cola or coca-cola or gingerale or ginger ale or pepsi or root beer* or "Dr. Pepper" or "7up").tw,kf. (1755)
91 (softdrink* or soft drink? or sodapop* or soda pop?).tw,kf. (3754)
92 (smoothy or smoothie*).tw,kf. (132)
93 (gatorade? or powerade? or "red bull").tw,kf. (261)
94 (almondshake? or almond shake? or dairyshake? or dairy shake? or milkshake? or milk shake? or soyshake? or soy shake?).tw,kf. (214)
95 (hot adj (chocolate or cocoa)).tw,kf. (29)
96 (cappuccino? or expresso? or latte?).tw,kf. (275251)
97 exp Sweetening Agents/ (226499)
98 (pre-sweet* or presweet* or pre-sugar* or presugar* or sweetener? or sugar substitute? or sugar-sweeten*).tw,kf. (7149)
99 or/14-98 (4803731)
100 exp Vitamins/ (319315)
101 vitamin$1.tw,kf. (217403)
102 Orthomolecular Therapy/ (197)
103 (orthomolecular or megavitamin* or mega-vitamin*).tw,kf. (227)
104 (multivitamin$1 or multi-vitamin$1 or multimineral$1 or multi-mineral$1 or nutriceutical$1 or nutraceutical$1).tw,kf. (10962)
105 (megados* or mega dosage* or mega dose* or mega dosi* or high dosage* or high dose* or high dosi* or large dosage* or large dose* or large dosi* or unorthodox or unconventional or high daily intake$1 or higher concentration$1).ti. (32693)
106 or/100-105 (440211)
107 (medium chain triacylglycerol$1 or medium chain triglyceride$1).tw,kf. (2690)
108 *Phosphatidylserines/ or phosphatidylserine$1.tw,kf. (15521)
109 gamma-oryzanol.tw,kf. (330)
110 exp *Fatty Acids/ or fatty acid$1.tw,kf. (387430)
111 exp Amino Acids/ae, ad, de, dt, to, ph, pk, th (89728)
112 exp Alanine/ or Arginine/ or Asparagine/ or exp Aspartic Acid/ or exp Cysteine/ or exp Glutamic Acid/ or exp Glutamine/ or exp Glycine/ or exp Histidine/ or Isoleucine/ or Leucine/ or exp Lysine/ or exp Methionine/ or exp Phenylalanine/ or exp Proline/ or exp Serine/ or exp Tyrosine/ or exp Tryptophan/ or exp Threonine/ or exp Valine/ (518854)
113 (amino acid$1 or alanine or "L-alanine" or arginine or "L-arginine" or asparagine or aspartic acid$1 or cysteine or glutamate or glutamic acid$1 or glutamine or histidine or isoleucine or leucine or lysine or methionine or phenylalanine or proline or serine or threonine or tryptophan or tyrosine or valine).tw,kf. (1197965)

114 Lactobacillus acidophilus/ or (lactobacilla or lactobacillus).tw,kf. (31001)
115 Anthocyanins/ or (anthocyanin$1 or anthocyanidin$1).tw,kf. (12248)
116 exp *Antioxidants/ or (antioxidant$1 or anti-oxidant$1).ti. (282488)
117 Apigenin/ or apigenin$1.tw,kf. (4609)
118 Arachidonic Acid/ or arachidonic acid$1.tw,kf. (42130)
119 beta Carotene/ or beta carotene.tw,kf. (15245)
120 Carotenoids/ or (carotenoid$1 or carotene$1).tw,kf. (36794)
121 Bifidobacterium/ or bifidobacterium.tw,kf. (9202)
122 Biotin/ or biotin.tw,kf. (32745)
123 Saccharomyces cerevisiae/ or saccharomyces cerevisiae.tw,kf. (118644)
124 Boron/ or boron.tw,kf. (17983)
125 Bromelains/ or bromelain?.tw,kf. (2032)
126 caffeic acid$1.tw,kf. (5984)
127 Caffeine/ or caffeine.tw,kf. (33856)
128 Calcium/ or calcium.tw,kf. (495559)
129 Catechin/ or catechin.tw,kf. (13299)
130 Epicatechin/ or epicatechin.tw,kf. (11768)
131 exp chitin/ or (chitin or chitosan).tw,kf. (37716)
132 Chlorogenic Acid/ or chlorogenic acid.tw,kf. (4935)
133 Choline/ or choline.tw,kf. (44365)
134 Chromium/ or chromium.tw,kf. (29652)
135 cinnamic acid$1.tw,kf. (2894)
136 cirsimaritin.tw,kf. (85)
137 Cobalt/ or cobalt.tw,kf. (44066)
138 ("coenzyme q10" or "co-enzyme q10" or coq10).tw,kf. (3914)
139 Copper/ or copper.tw,kf. (125030)
140 coumarin.tw,kf. (11128)
141 (allyl sulfide$1 or diallyl sulfide$1).tw,kf. (482)
142 eriodictyol.tw,kf. (453)
143 Eugenol/ or eugenol.tw,kf. (4829)
144 Fish Oils/ or Cod Liver Oil/ or exp Fatty Acids, Omega-3/ or (fish oil? or "omega-3" or O3FA).tw,kf. (37060)
145 ferulic acid$1.tw,kf. (4990)
146 fisetin.tw,kf. (795)
147 exp Folic Acid/ or (folic acid$1 or folate).tw,kf. (56373)
148 exp Leucovorin/ or (folinic acid$1 or leucovorin).tw,kf. (13223)
149 gamma-Linolenic Acid/ or (gammalinolenic or gamma linolenic).tw,kf. (2717)
150 Hesperidin/ or hesperidin.tw,kf. (2190)
151 exp Inositol/ or inositol.tw,kf. (44508)
152 Inulin/ or inulin.tw,kf. (11330)
153 Iodine/ or iodine.tw,kf. (61226)
154 Iron/ or iron.tw,kf. (209583)
155 isoquercitrin.tw,kf. (731)
156 isorhamnetin.tw,kf. (1213)
157 (k?empferol or k?empferitrin or kombucha).tw,kf. (5310)
158 larch arabinogalactan.tw,kf. (33)
159 exp Phosphatidylcholines/ or (phosphatidylcholine or phosphatidyl choline or lecithin$1).tw,kf. (60805)

160 Linoleic Acid/ or (linoleic acid$1 or linoleate).tw,kf. (21710)
161 exp Xanthophylls/ or (lutein or xanthophyll).tw,kf. (9364)
162 Luteolin/ or luteolin$1.tw,kf. (4225)
163 lycopene.tw,kf. (4920)
164 Magnesium/ or magnesium.tw,kf. (100419)
165 Manganese/ or manganese.tw,kf. (46434)
166 Trace Elements/ or (micronutrient$1 or micro-nutrient$1 or trace element$1 or trace mineral$1).tw,kf. (42489)
167 Minerals/ or mineral$1.tw,kf. (134216)
168 Molybdenum/ or molybdenum.tw,kf. (16138)
169 (dimethyl sulfone or methylsulfonylmethane).tw,kf. (235)
170 myricetin.tw,kf. (1909)
171 Niacin/ or (niacin or nicotinic acid$1).tw,kf. (16923)
172 Nickel/ or nickel.tw,kf. (45338)
173 Oleanolic Acid/ or oleanolic acid$1.tw,kf. (4574)
174 Papain/ or papain.tw,kf. (10618)
175 Phosphorus/ or phosphorous.tw,kf. (46221)
176 Phytosterols/ or (phytosterol$1 or phyto-sterol$1).tw,kf. (4754)
177 Potassium/ or potassium.tw,kf. (196249)
178 proanthocyanidin.tw,kf. (1610)
179 procyanidin.tw,kf. (1437)
180 Pyridoxine/ or (pyridoxine$1 or "vitamin b 6" or "vitamin b6").tw,kf. (14317)
181 Pyruvic Acid/ or (pyruvic acid$1 or pyruvate).tw,kf. (43641)
182 Quercetin/ or quercetin.tw,kf. (18830)
183 (retinol acetate or retinyl acetate or retinol palmitate or retinyl palmitate).tw,kf. (2020)
184 exp Retinoids/ or (retinoid$1 or retinoic or retinal).tw,kf. (222413)
185 Riboflavin/ or riboflavin.tw,kf. (12943)
186 Rutin/ or (rutin or rutine or rutoside).tw,kf. (6673)
187 Selenium/ or selenium.tw,kf. (32745)
188 (sesamol or sesamolin).tw,kf. (362)
189 Silicon/ or silicon.tw,kf. (52189)
190 Sodium/ or ((dietary or intake) adj1 sodium).tw,kf. (109910)
191 stevioside.tw,kf. (474)
192 (sulforafan or sulforaphane).tw,kf. (2071)
193 Sulfur/ or (sulfur or sulphur).tw,kf. (66454)
194 exp Tannins/ or (tannin$1 or tannic).tw,kf. (18798)
195 taxifolin.tw,kf. (622)
196 (theaflavin or thearubigin).tw,kf. (337)
197 exp Thiamine/ or thiamin$1.tw,kf. (17352)
198 Tin/ or tin.tw,kf. (17444)
199 Tocopherol/ or alpha Tocopherol/ or (tocopherol or "vitamin e").tw,kf. (42674)
200 Tocopherol/ or tocopherol$1.tw,kf. (21422)
201 Vanadium/ or vanadium.tw,kf. (9686)
202 Vanillic Acid/ or vanillic acid$1.tw,kf. (1860)
203 exp "Vitamin B 12"/ or (cyanocobalamin or "vitamin b 12" or "vitamin b12").tw,kf. (30328)
204 exp Ascorbic Acid/ or (ascorbic acid$1 or "vitamin c").tw,kf. (64130)
205 exp "Vitamin d"/ or (ergocalciferol$1 or "vitamin d" or "vitamin d 2" or "vitamin d2" or "vitamin d 3" or "vitamin d3").tw,kf. (85037)

206 exp "Vitamin K"/ or "vitamin k".tw,kf. (25624)
207 exp "Vitamin A"/ or "vitamin a".tw,kf. (53563)
208 exp "Vitamin E"/ or "vitamin e".tw,kf. (42896)
209 provitamin$1.tw,kf. (998)
210 exp Whey Proteins/ or (whey protein$1 or protein powder?).tw,kf. (16949)
211 Zinc/ or zinc.tw,kf. (134971)
212 Saccharum/ or (saccharum officinarum or sugarcane or sugar cane).tw,kf. (6460)
213 exp Citrus/ or (citrus or grapefruit$1 or orange$1 or tangerine$1).tw,kf. (42851)
214 Lycopersicon esculentum/ or (lycopersicon esculentum or tomato*).tw,kf. (26566)
215 Ubiquinone/ or ubiquinone.tw,kf. (12778)
216 (dietary fat$1 and unsaturated fatty acid$1).tw,kf. (265)
217 exp Prunus/ or (apricot$ or prune$1 or prunus).tw,kf. (8792)
218 Hordeum/ or (hordeum or barley$1).tw,kf. (18897)
219 Blueberry Plant/ or blueberr*.tw,kf. (2276)
220 exp Citrullus/ or (citrullus or watermelon$1).tw,kf. (2210)
221 Fabaceae/ or (bean or beans or leguminosae or legume?).tw,kf. (44204)
222 Phaseolus/ or (common bean$1 or french bean$1 or kidney bean$1 or phaseolus).tw,kf. (8726)
223 Psidium/ or guava$1.tw,kf. (958)
224 Humulus/ or (hops or humulus lupulus).tw,kf. (2280)
225 Juniperus/ or juniper*.tw,kf. (1425)
226 Mango/ or (mango or mangoes).tw,kf. (2071)
227 Olive Oil/ or olive oil$1.tw,kf. (10987)
228 Carica/ or (carica or papaya$1).tw,kf. (2529)
229 Plantago/ or plaintain$1.tw,kf. (851)
230 Helianthus/ or (helianthus annuus or sunflower$1).tw,kf. (8465)
231 or/107-230 (4308432)
232 106 and 231 (381891)
233 (selenium adj2 vitamin e adj5 select).tw,kf. (87)
234 (prebiotic$1 or probiotic$1 or synbiotic$1).tw,kf. (29426)
235 Ellagic Acid/ad, tu (185)
236 (ellagic acid and ("therapeutic use" or antiinflammator* or anti-inflammator* or oral administration*)).tw,kf. (289)
237 (sulfinic acid$1 and allicin).tw,kf. (4)
238 (glyconutrient$ or glyco-nutrient$).tw,kf. (11)
239 Euterpe/ or (acai or euterpe).tw,kf. (465)
240 citrus pectin$1.tw,kf. (404)
241 Curcumin/ or curcumin.tw,kf. (14769)
242 (oleum and menth* and piperitae).tw,kf. (4)
243 mushroom*.tw,kf. (15416)
244 Ipomoea batatas/ or purple sweet potato anthocyanin.tw,kf. (1073)
245 (pumpkin seed$1 and (therapy or therapies or treatment)).tw,kf. (81)
246 (rice or rice bran).tw,kf. (53255)
247 (wssf or water-soluble soybean fiber or water-soluble soybean fibre).tw,kf. (6)
248 Phytosterols/ or (phytosterol$1 or phyto-sterol$1).tw,kf. (4754)
249 Soybeans/ or (soybean$1 or soy bean$1 or glycine max).tw,kf. (45210)
250 exp Isoflavones/ or (isoflavone$1 or ipriflavone$1 or genistein or homoisoflavone$1).tw,kf. (24172)

251 (exp Antioxidants/ or (antioxidant$1 or anti-oxidant$1 or consumption or treatment$1 or therapy or therapies or therapeutic* or prevention*).tw,kf.) and (Flavonoids/ or (flavonoid$1 or flavinoid$1 or bioflavonoid$1 or bioflavonoid$1 or bio-flavonoid$1 or bio-flavonoid$1 or bioflavinoid$1 or bioflavinoid$1 or bio-flavinoid$1 or bio-flavinoid$1).tw,kf.) (36844)
252 ("coenzyme q10" or "coenzyme q" or coq10).ti. (3114)
253 *Thioctic Acid/ or (alpha-lipoic acid$1 or lipoic acid$1 or thioctic acid$1).tw,kf. (5365)
254 resveratrol.tw,kf. or resveratrol.rn. (12912)
255 Medicago Sativa/ or (alfalfa or medicago sativa).tw,kf. (8933)
256 Cynara scolymus/ or (artichoke or cynara scolymus).tw,kf. (1104)
257 Momordica charantia/ or (momordica charantia or bitter melon$1).tw,kf. (1045)
258 Prunus avium/ or black cherr$3.tw,kf. (355)
259 Rubus/ or (blackberr$3 or raspberr$3 or rubus fruticosus or rubus idaeus).tw,kf. (2731)
260 Vaccinium/ or Blueberry Plant/ or (bilberr$3 or blueberr$3 or vaccinium).tw,kf. (3464)
261 Carum/ or (carum or caraway).tw,kf. (417)
262 Capsicum/ or (capsicum or cayenne).tw,kf. (4777)
263 Apium graveolens/ or (celery or celeriac or apium graveolens).tw,kf. (1294)
264 Matricaria/ or matricaria.tw,kf. (600)
265 Chamomile/ or chamomile.tw,kf. (964)
266 Chicory/ or (chicory or cichorium intybus).tw,kf. (982)
267 Cinnamomum zeylanicum/ or (cinnamomum zeylanicum or cinnamomum verum or cinnamon).tw,kf. (2504)
268 Eugenia/ or Syzygium/ or (clove or cloves or syzygium aromaticum).tw,kf. (2341)
269 Cocos/ or (cocoanut$1 or coconut$1).tw,kf. (4714)
270 Cola/ or (cola acuminata or cola nitida).tw,kf. (139)
271 Coriandrum/ or (coriandrum or coriander).tw,kf. (736)
272 Vaccinium macrocarpon/ or (cranberry or vaccinium macrocarpon).tw,kf. (1561)
273 Phoeniceae/ or (phoenix dactylifera or date palm).tw,kf. (1121)
274 Anethum graveolens/ or (dill or anethum graveolens).tw,kf. (478)
275 Foeniculum/ or (foeniculum or fennel).tw,kf. (821)
276 Trigonella/ or (trigonella or fenugreek or foenumgraecum or foenum graecum or trigonella).tw,kf. (1261)
277 Flax/ or (flax or linseed$1 or linum or flaxseed$1).tw,kf. (5292)
278 Garlic/ or (garlic or allium sativum).tw,kf. (6604)
279 Ginger/ or (ginger or zingiber* or zinziber*).tw,kf. (4829)
280 Lycium/ or (goji or lycium barbarum or wolfberr*).tw,kf. (943)
281 Vitis/ or (grape$1 or raisin$1 or vitis).tw,kf. (64556)
282 Citrus paradisi/ or (grapefruit* or citrus paradisi or citrus x paradisi).tw,kf. (2213)
283 Hibiscus/ or hibiscus sabdariffa.tw,kf. (881)
284 exp Ocimum/ or (basil or ocimum basilicum or ocimum tenuiflorum).tw,kf. (1761)
285 Armoracia/ or (armoracia or horseradish* or horse radish*).tw,kf. (22657)
286 (mung bean$1 or vigna radiata).tw,kf. (2012)
287 exp Nigella/ or (black cumin or nigella sativa).tw,kf. (1468)
288 Myristica fragrans/ or (nutmeg or myristica fragrans).tw,kf. (576)
289 Olea/ or (olive tree$1 or olea).tw,kf. (4107)
290 Citrus sinensi/ or (sweet orange$1 or citrus sinensis).tw,kf. (2797)
291 Origanum/ or (oregano or origanum vulgare).tw,kf. (1495)
292 Petroselinum/ or (petroselinum crispum or parsley).tw,kf. (1108)
293 Mentha piperita/ or (mentha piperita or "mentha x piperita" or peppermint).tw,kf. (1561)

294 (ispagula or psyllium).tw,kf. (738)
295 exp Ganoderma/ or (reishi or ganoderma lucidum or ganoderma tsugae).tw,kf. (2183)
296 Aspalathus/ or (aspalathus linearis or rooibos).tw,kf. (266)
297 Rosmarinus/ or (rosmarinus officinalis or rosemary).tw,kf. (2042)
298 Salvia officinalis/ or (salvia officinalis or sage).tw,kf. (4053)
299 Shiitake Mushrooms/ or (lentinula edodes or shiitake mushroom$1).tw,kf. (912)
300 Spirulina/ or spirulina.tw,kf. (2010)
301 Stevia/ or stevia.tw,kf. (787)
302 (origanum marjorana or marjoram).tw,kf. (212)
303 (citrus reticulata or mandarin).tw,kf. (3261)
304 Camellia sinensis/ or camellia sinensis.tw,kf. (3636)
305 Thymus Plant/ or (thyme or thymus vulgaris).tw,kf. (2356)
306 Curcuma/ or (curcuma longa or turmeric or tumeric).tw,kf. (4990)
307 Mustard Plant/ or white mustard$1.tw,kf. (2151)
308 or/233-307 (406369)
309 (Iodine/ or exp "Vitamin D"/ or exp Folic Acid/ or "Vitamin B 12"/ or exp Fatty Acids, Omega-3/) and (diet or diets or dietary or supplementation).ti. (13055)
310 (exp Disease/ or exp Oxidative Stress/) and exp dietary supplements/ and (humans/ or clinical trial.pt.) (1798)
311 Soil Pollutants/ or exp Pesticides/ or exp Invertebrates/ or Animal Husbandry/ or soil.ti. or (insect or insecticid* or entomology or biofuel*).tw,kf. (865146)
312 (309 or 310) not 311 (14623)
313 99 or 232 or 308 or 312 (5101892)
314 13 and 313 (30653)
315 (comment or editorial or letter or news or newspaper article).pt. (2021087)
316 314 not 315 [OPINION PIECES REMOVED] (30375)
317 exp Animals/ not Humans/ (4680951)
318 316 not 317 [ANIMAL-ONLY REMOVED] (24625)

****
